# BDNF Overexpression Enhances the Preconditioning Effect of Brief Episodes of Hypoxia, Promoting Survival of GABAergic Neurons

**DOI:** 10.1007/s12264-020-00480-z

**Published:** 2020-03-27

**Authors:** M. V. Turovskaya, S. G. Gaidin, M. V. Vedunova, A. A. Babaev, E. A. Turovsky

**Affiliations:** 1 Institute of Cell Biophysics of the Russian Academy of Sciences, Federal Research Center “Pushchino Scientific Center for Biological Research of the Russian Academy of Sciences”, Pushchino, Russia; 2 Institute of Biology and Biomedicine, Lobachevsky State University of Nizhny Novgorod, Nizhny Novgorod, Russia

**Keywords:** Hypoxia, Neuron, BDNF, Preconditioning, Calcium, Receptors

## Abstract

Hypoxia causes depression of synaptic plasticity, hyperexcitation of neuronal networks, and the death of specific populations of neurons. However, brief episodes of hypoxia can promote the adaptation of cells. Hypoxic preconditioning is well manifested in glutamatergic neurons, while this adaptive mechanism is virtually suppressed in GABAergic neurons. Here, we show that brain-derived neurotrophic factor (BDNF) overexpression in neurons enhances the preconditioning effect of brief episodes of hypoxia. The amplitudes of the NMDAR- and AMPAR-mediated Ca^2+^ responses of glutamatergic and GABAergic neurons gradually decreased after repetitive brief hypoxia/reoxygenation cycles in cell cultures transduced with the (AAV)-Syn-BDNF-EGFP virus construct. In contrast, the amplitudes of the responses of GABAergic neurons increased in non-transduced cultures after preconditioning. The decrease of the amplitudes in GABAergic neurons indicated the activation of mechanisms of hypoxic preconditioning. Preconditioning suppressed apoptotic or necrotic cell death. This effect was most pronounced in cultures with BDNF overexpression. Knockdown of BDNF abolished the effect of preconditioning and promoted the death of GABAergic neurons. Moreover, the expression of the anti-apoptotic genes Stat3, Socs3, and Bcl-xl substantially increased 24 h after hypoxic episodes in the transduced cultures compared to controls. The expression of genes encoding the pro-inflammatory cytokines IL-10 and IL-6 also increased. In turn, the expression of pro-apoptotic (Bax, Casp-3, and Fas) and pro-inflammatory (IL-1β and TNFα) genes decreased after hypoxic episodes in cultures with BDNF overexpression. Inhibition of vesicular BDNF release abolished its protective action targeting inhibition of the oxygen-glucose deprivation (OGD)-induced [Ca^2+^]_i_ increase in GABAergic and glutamatergic neurons, thus promoting their death. Bafilomycin A1, Brefeldin A, and tetanus toxin suppressed vesicular release (including BDNF) and shifted the gene expression profile towards excitotoxicity, inflammation, and apoptosis. These inhibitors of vesicular release abolished the protective effects of hypoxic preconditioning in glutamatergic neurons 24 h after hypoxia/reoxygenation cycles. This finding indicates a significant contribution of vesicular BDNF release to the development of the mechanisms of hypoxic preconditioning. Thus, our results demonstrate that BDNF plays a pivotal role in the activation and enhancement of the preconditioning effect of brief episodes of hypoxia and promotes tolerance of the most vulnerable populations of GABAergic neurons to hypoxia/ischemia.

**Electronic supplementary material:**

The online version of this article (10.1007/s12264-020-00480-z) contains supplementary material, which is available to authorized users.

## Introduction

Many cardiovascular diseases such as angina, myocardial infarction, cardiac insufficiency, and peripheral arteriolar constriction are followed by hypoxia. Hypoxic conditions and ischemia are caused by arterial occlusion and anatomical capillary rarefaction due to hypertension. Hypoxia also occurs during rapid tissue growth during organogenesis or cancer tumor formation, chronic inflammation, and altitude sickness [1].

Oxygen consumption by the mammalian brain is very high, accounting for 20% of body oxygen consumption [2]. This metabolic peculiarity makes brain cells particularly sensitive to hypoxia. Acute episodes of hypoxia depress synaptic activity, while prolonged exposure to hypoxia leads to the death of neurons [3]. Episodes of ischemia that last > 2 min can lead to the death of neurons [4], especially GABAergic neurons without Ca^2+^-binding proteins [5, 6].

On the contrary, positive effects of brief hypoxia have also been reported. For instance, low oxygen tension promotes the proliferation of endothelial cells [7] and angiogenesis [8], thus ameliorating tissue hypoxia. Such positive effects are tightly coupled to the phenomenon of hypoxic preconditioning (HP), which is considered to be an efficient approach to reducing the vulnerability of tissues and organs to prolonged hypoxia/ischemia-reoxygenation episodes. The approach consists of a single [9] or repetitive brief episodes of hypoxia followed by reoxygenation [10, 11]. HP can be divided into two types—rapid and delayed—according to the time interval between the stimulus and the development of adaptation. Rapid HP develops in the range from minutes to some hours after sublethal hypoxia. This type of HP is mediated by changes in the conductivity of ion channels and the phosphorylation of proteins and their post-translational modification [12–15]. On the contrary, delayed HP requires gene expression and *de novo* protein synthesis. The effects of delayed HP can be detected some hours or days after the stimulus. Delayed HP involves the activation of genes that promote tolerance of the brain to ischemia, suppression of the mechanisms of cell damage, and enhancement of the mechanisms of cell survival [16].

HP for neuroprotection was first used in 1986 [12]. Brain slices and primary cell cultures from different brain regions are used as *in vitro* models of HP in brain research [17, 18]. It has been shown that a single 2-min and three repetitive 1-min episodes of anoxia (in slices of the olfactory cortex and hippocampus, respectively) increase the tolerance of cells to prolonged anoxia, inhibit the depression of evoked potentials, and suppress global Ca^2+^ increases. Interestingly, a moderate increase in intracellular Ca^2+^ concentration ([Ca^2+^]_i_) is necessary for the induction of HP in both models [19]. We have previously described a cellular model that includes three brief (3-min) episodes of hypoxia followed by three 10-min reoxygenation periods. This model allows detection of the development of HP in neurons by changes in the amplitudes of Ca^2+^ responses to the application of agonists. It is also possible to detect post-hypoxic hyperexcitation by the appearance of spontaneous Ca^2+^ signals, which can promote the death of some neuronal populations during reoxygenation [20].

The role of neurotrophic factors in the protection of cells against ischemia and activation of the mechanisms of preconditioning has been studied in the past few years. Brain-derived neurotrophic factor (BDNF) is the most common neurotrophin in the brain, and its expression is affected by many external and internal factors. Altered BDNF expression occurs under ischemia, hypoxia, brain trauma, and various stresses. It regulates neurotransmission and cell survival *via* the activation of different receptors [21]. We have previously shown that BDNF overexpression alters the expression of genes that regulate neurotransmission, inflammation, and apoptosis, thus protecting hippocampal cells against death under oxygen-glucose deprivation (OGD) and glutamate toxicity [22].

It has been shown that preconditioning of rats with three episodes of moderate hypoxia evokes an increase in the BDNF level one day later and promotes their tolerance to traumatic injury. HP stimulates BDNF expression in a long-term manner in the neocortex and hippocampus in a model of post-traumatic stress disorder-associated anxiety [23], however, the protective effects of BDNF overexpression on different populations of neurons have not yet been investigated, while the mechanisms and signaling pathways involved in HP formation in GABAergic neurons remain unclear. Taking into account the peculiar vulnerability of GABAergic neurons to hypoxia and their role in the regulation of neuronal network activity, it can be concluded that studying the mechanisms of HP formation in this population of neurons is an important issue. Thus, the present study was designed to investigate the effects of BDNF overexpression on the activation and enhancement of HP in glutamatergic and GABAergic neurons.

## Materials and Methods

### Animals and Reagents

All animal studies were performed in accordance with legal requirements and were approved by the Animal Ethics Committees of the Institute of Cell Biophysics, Russian Academy of Sciences. Pregnant female Sprague-Dawley rats were housed in the animal facility of the Institute of Cell Biophysics, Russian Academy of Sciences, at 25 ± 3°C with a 12-h light/dark cycle and free access to food and water.

Reagents included 5-fluorowillardiine, N-methyl-*D*-aspartate (NMDA), bafilomycin A1 (BafA1), brefeldin A (BFA), sodium dithionite (Tocris Bioscience, Bristol, UK); KCl, tetanus toxin (TeNT), paraformaldehyde, poly(ethyleneimine), adenosine 5′-triphosphate disodium salt hydrate (ATP) (Sigma-Aldrich, St. Louis, MO); Fura-2AM, Hoechst 33342, propidium iodide (Thermo Fisher Scientific, Waltham, MA); Neurobasal-A medium, B-27 supplement, trypsin (1%) (Life Technologies, Grand Island, NY); donkey serum, mouse anti-NeuN antibodies, chicken anti-BDNF antibodies, donkey anti-rabbit Alexa Fluor-488- and Alexa Fluor-555- conjugated antibodies, donkey anti-mouse Alexa Fluor-647- conjugated antibodies, and donkey anti-chicken Alexa Fluor-488- conjugated antibodies (Abcam, Cambridge, UK).

### Preparation of Mixed Hippocampal Neuroglial Cell Cultures

Cell cultures were prepared as described in detail previously [5]. Briefly, 0–1 day-old pups were euthanized by halothane overdose and decapitated. The extracted hippocampus was washed with Mg^2+^- and Ca^2+^-free Versene solution and minced with scissors. Then, the tissue fragments were digested in 1% trypsin for 10 min at 37 °C and washed twice with cold Neurobasal-A medium. The trypsinized tissue was gently triturated with a pipette, and the debris was then carefully removed with a pipette tip. The cell suspension was seeded on polyethyleneimine-coated glass coverslips and grown for 10–12 days in Neurobasal-A medium supplemented with 2% B-27 and 0.5 mmol/L glutamine.

References [5, 25, 26] BafA1, BFA, and TeNT were added to the medium under sterile conditions in experiments with 24-h pre-incubation. Then, the cultures were washed with Hank`s balanced salt solution (HBSS) containing inhibitors and used in experiments.

### Induction of BDNF Overexpression in Neurons

Mixed neuroglial hippocampal cell cultures were transduced with the (AAV)-Syn-BDNF-EGFP virus vector to induce BDNF overexpression in neurons. The structure of this adeno-associated viral construct was described previously [24]. To achieve selective BDNF overexpression in neurons, the human synapsin (hSyn) promoter sequence was incorporated into the vector. The (AAV)-Syn-BDNF-EGFP construct was added to cultures at 4–5 days *in vitro* (DIV) (construct dilution 1:125). BDNF overexpression occurred mainly in hippocampal neurons 24 h after transduction (Fig. S1) and was maintained until 10–12 DIV.

### Ca^2+^ Fluorescence Measurements

To assess the changes in [Ca^2+^]_i_, hippocampal cell cultures were loaded with Fura-2 (4 µmol/L, 40-min incubation at 37°C). The cells were stained with the probe dissolved in HBSS composed of (in mmol/L): 156 NaCl, 3 KCl, 2 MgSO_4_, 1.25 KH_2_PO_4_, 2 CaCl_2_, 10 glucose, and 10 HEPES, pH 7.4. To measure [Ca^2+^]_i_, we used a system based on an inverted motorized microscope (Leica Microsystems, Wetzlar, Germany; DMI6000B) with a high-speed monochrome CCD-camera Hamamatsu C9100 (Hamamatsu Photonics, Hamamatsu City, Japan). For excitation and registration of Fura-2 fluorescence, we used the FU-2 filter set (Leica Microsystems, Wetzlar, Germany) with excitation filters (BP340/30 and BP387/15), a beam-splitter (FT-410), and an emission filter (BP510/84). An illuminator with a high-pressure mercury lamp (Leica EL6000) was used as the source of excitation. To distinguish neurons from astrocytes, we briefly applied 35 mmol/L KCl and 10 µmol/L ATP before the main experiments. This method has been described in detail in our previous report References [5, 25, 26]. Briefly, KCl depolarizes excitable cells that contain a wide range of voltage-gated cation channels. The KCl-induced depolarization promotes the opening of voltage-gated Ca^2+^ channels (predominantly L-type channels) in neurons. The KCl-induced increase in [Ca^2+^]_i_ is easily detected with Ca^2+^-sensitive fluorescent probes such as Fura-2 and Fluo-4. The Ca^2+^ responses of neurons were characterized by rapid rising and slow decay phases. The conductivity and density of cation channels in astrocytes are insufficient to evoke a high-amplitude Ca^2+^ response to KCl. In turn, the ATP-induced biphasic Ca^2+^ response, which is mediated by ionotropic and metabotropic purinergic receptors, occurs mainly in astrocytes due to the rich repertoire of P2X and P2Y receptors, while the response of neurons to ATP is negligible. The ratio of neurons to glial cells was determined by immunostaining with antibodies against NeuN. This ratio was 1:3 for our protocol of cell culture preparation. All the Ca^2+^ signals are presented as the 340/380 ratio of Fura-2 fluorescence.

### Techniques for Modeling Brief Hypoxic Episodes and Prolonged Oxygen-glucose Deprivation (OGD)

To model hypoxic conditions, we used HBSS with a low concentration of dissolved O_2_. The HBSS was purged for 15 min with argon in a special hermetic system to displace O_2_. O_2_ tension was measured with a Clark electrode and reached 50–60 mmHg, corresponding to moderate hypoxia. Each hypoxia/reoxygenation cycle consisted of a 3-min episode of hypoxia when the hypoxic medium was added to the experimental chamber with the hippocampal cell culture and a 10-min reoxygenation episode when the cultures were washed with HBSS containing the normal concentration of dissolved O_2_. α-Amino-3-hydroxy-5-methylisoxazole-4-propionic acid receptor (AMPAR) or NMDAR agonists were applied briefly (30 s) after each hypoxia/reoxygenation cycle. The drugs were added at 10 L/min using a specially-designed perfusion system. The volume of medium in the experimental chamber was 500 µL, and excess liquid was drained with a water-jet pump. Inlet and outlet pipes were installed on the opposite sides of the experimental chamber. Using a colored solution, we determined that complete replacement of the bathing solution in the chamber occurred 20–30 s after the start of perfusion. This system allowed the short-term and long-term application of drugs. Control experiments established that the responses of cells to mechanical stimulation caused by the fluid flow during drug application were negligible or absent.

The development of the preconditioning effect was estimated by the changing amplitudes of NMDA-induced Ca^2+^ responses after hypoxia/reoxygenation cycles. The amplitudes of the Ca^2+^ responses to NMDA application before and after three episodes of hypoxia/reoxygenation were calculated for all analyzed neurons, and the values were presented in a coordinate plane (X-axis, amplitude before hypoxia; Y-axis, amplitude after hypoxia). Then the data points were approximated by linear regression. The slopes of the regression lines were used to estimate the preconditioning effect of hypoxic episodes. A decrease in the slope indicated the development of preconditioning. To investigate the protective effects of HP, the cultures were returned to a CO_2_ incubator after three episodes of hypoxia/reoxygenation and then used in OGD experiments 24 h after preconditioning.

In turn, to model ischemia-like conditions, glucose was displaced by an equivalent quantity of sucrose (HBSS without glucose). O_2_ tension in the medium reached 30–40 mmHg and the OGD lasted for 40 min. To prevent contact of the medium with atmospheric O_2_, we used a constant argon feed into the experimental chamber during brief episodes of hypoxia or prolonged OGD. In some experiments (Fig. 4), the cultures were returned to the CO_2_ incubator for 24 h after OGD, and the ischemic medium was replaced by standard culture medium.

To create OGD conditions in release experiments, complete HBSS was replaced by glucose-free HBSS (glucose replaced by an osmotically equivalent concentration of sucrose) 1.5–2 min before the experiment. Then, the O_2_-scavenger sodium dithionite (30 µg/mL) was added to the medium during the measurement of EGFP fluorescence to remove dissolved O_2_. This dose of sodium dithionite removes O_2_ without changing the pH, which has been established using Blood Gas Electrolyte Analyzer (Siemens Healthineers, Erlangen, Germany).

### Immunocytochemistry

Coverslips with hippocampal cell cultures were mounted in the experimental chamber. A marker grid was plotted on the bottom of each coverslip. The chamber was placed on the microscope stage, and a grid-bordered area was randomly selected for fluorescent Ca^2+^ imaging. Then the cells were fixed and stained with antibodies according to the previously-described protocol [22]. Briefly, the cells were rinsed with Ca^2+^- and Mg^2+^-free phosphate-buffered saline (PBS) and fixed for 20 min in 4% paraformaldehyde and 0.25% glutaraldehyde diluted in PBS. After that, the cells were rinsed thrice in ice-cold PBS and permeabilized with 0.1% Triton X-100. Then, the cells were incubated for 30 min with 10% donkey serum (in PBS) to block non-specific binding of the secondary antibodies and stained overnight at 4°C with primary antibodies diluted in 1% donkey serum. We used mouse anti-NeuN antibodies (1:200; Abcam) to identify neurons and rabbit anti-glutamate decarboxylase 65/67 (GAD65/67) antibodies (1:500; Abcam) to discriminate GABAergic neurons. Chicken anti-BDNF antibodies (1:150; Abcam) were used to determine the level of BDNF in cells. The cells were rinsed thrice with PBS after incubation with the primary antibodies and stained with secondary antibodies. We used secondary donkey anti-rabbit Alexa Fluor-488- or Alexa Fluor-555-conjugated (1:200; Abcam), donkey anti-mouse Alexa Fluor-647-conjugated (1:200; Abcam), and donkey anti-chicken Alexa Fluor-488-conjugated antibodies (1:200; Abcam). Fluorescence of the conjugated dyes was detected with a Leica TCS SP5 confocal microscope in the grid-bordered areas chosen for Ca^2+^ imaging. The confocal images of cell cultures stained with the antibodies were matched with the images of the same cultures captured during vital Ca^2+^ imaging. Thus, the combination of vital Ca^2+^ imaging and immunostaining allowed us to obtain data about Ca^2+^ dynamics in NeuN-positive or GAD65/67-positive cells.

### Assessment of BDNF Release

BDNF release in cultures transduced with the (AAV)-BDNF-EGFP construct was assessed as a decrease of EGFP fluorescence in the processes and somata of neurons using the Leica TCS SP5 confocal microscope. EGFP fluorescence was excited with a 488-nm argon laser and detected in the 505–575 nm range. High-resolution images were captured using a Leica HCX PL APO lambda blue 63.0 × 1.40 oil objective. Time-lapse series were recorded at a rate of 1 scan per 10 s. The depth of each optical slice was ~ 2 µm. To avoid phototoxicity, the laser power was set to the minimum (1%–4% of laser output).

### Cell Viability Test

Hoechst 33342 (2 µmol/L) and propidium iodide (1 µmol/L) were used to evaluate the number of dead cells in cultures before and after OGD. The cells were stained for 5 min with the probes diluted in HBSS and then rinsed in HBSS. The fluorescence of the probes was detected with an inverted fluorescence microscope (Zeiss Axio Observer Z1) using Filter Sets 01 and 20. Early and late apoptotic cells were discriminated using a previously-described method [22, 27]. Five different areas in each culture were analyzed. Each experimental group consisted of three cultures from different passages.

### Transfection with Small-interfering RNA (siRNA)

When confluence reached 40% (5 DIV), cells were transfected with siRNA against rat BDNF (Thermo Fisher Scientific) using Lipofectamine RNAiMax (Life Technologies, Grand Island, NY) according to the manufacturer’s instructions. After incubating hippocampal cells with siRNA, reagent mixtures in Opti-MEM (Life Technologies) containing 50 pmol/L of shBDNF were added and incubated for 6 h. Then the medium was changed and cells were incubated for an additional 48 h. The efficiency of knockdown was at least 85%–90% as confirmed by RT-PCR and immunostaining with anti-BDNF antibodies (Fig. S2).

### Extraction of RNA

The Mag Jet RNA Kit (Thermo Fisher Scientific) was used for the extraction of total RNA. The RNA quality was estimated by electrophoresis in the presence of 1 μg/mL ethidium bromide (2% agarose gel in Tris/borate/EDTA buffer). The concentration of the extracted RNA was determined with a NanoDrop 1000c spectrophotometer (Thermo Fisher Scientific, Wilmington, DE). The RevertAid H Minus First Strand cDNA Synthesis Kit (Thermo Fisher Scientific) was used for reverse transcription of total RNA.

### Real-time Polymerase Chain Reaction (RT-qPCR)

Each PCR was performed in a 25-μL mixture of 5 μL of qPCRmix-HS SYBR (Evrogen, Moscow, Russia), 1 μL (0.2 μmol/L) of the primer solution, 17 μL RNase-free water, and 1 μL cDNA. The Dtlite Real-Time PCR System (DNA-technology, Moscow, Russia) was used for amplification, which consisted of an initial 5-min denaturation at 95°C, 40 cycles of 30 s denaturation at 95°C, 20 s annealing at 60–62°C, and a 20 s extension step at 72°C. The final extension was for 10 min at 72°C. The sequences of the primers are listed in Table S1. All the sequences were designed with FAST PCR 5.4 and NCBI Primer-BLAST software. The data were analyzed with Dtlite software (DNA-technology). The expression of the studied genes was normalized to the gene encoding glyceraldehyde 3-phosphate dehydrogenase (GAPDH). Data were analyzed using Livak’s method [28].

### Statistical Analysis

All presented data were from at least three cultures from 2–3 different passages. *n* indicates the number of experiments. All values are given as the mean ± standard error (SE). The significance of differences between two groups was estimated with the paired *t*-test. Two-way or one-way analysis of variance (ANOVA) followed by the Tukey-Kramer *post-hoc* test was used for multiple group comparisons. The statistical tests were performed using GraphPad Prism 5 software (GraphPad Software, San Diego, CA).

## Results

### BDNF Overexpression Activates HP in GABAergic Hippocampal Neurons and Reduces the Amplitude of Ca^**2+**^ Responses by Changing the Expression of AMPAR and NMDAR Subunits

**Fig. 1:**
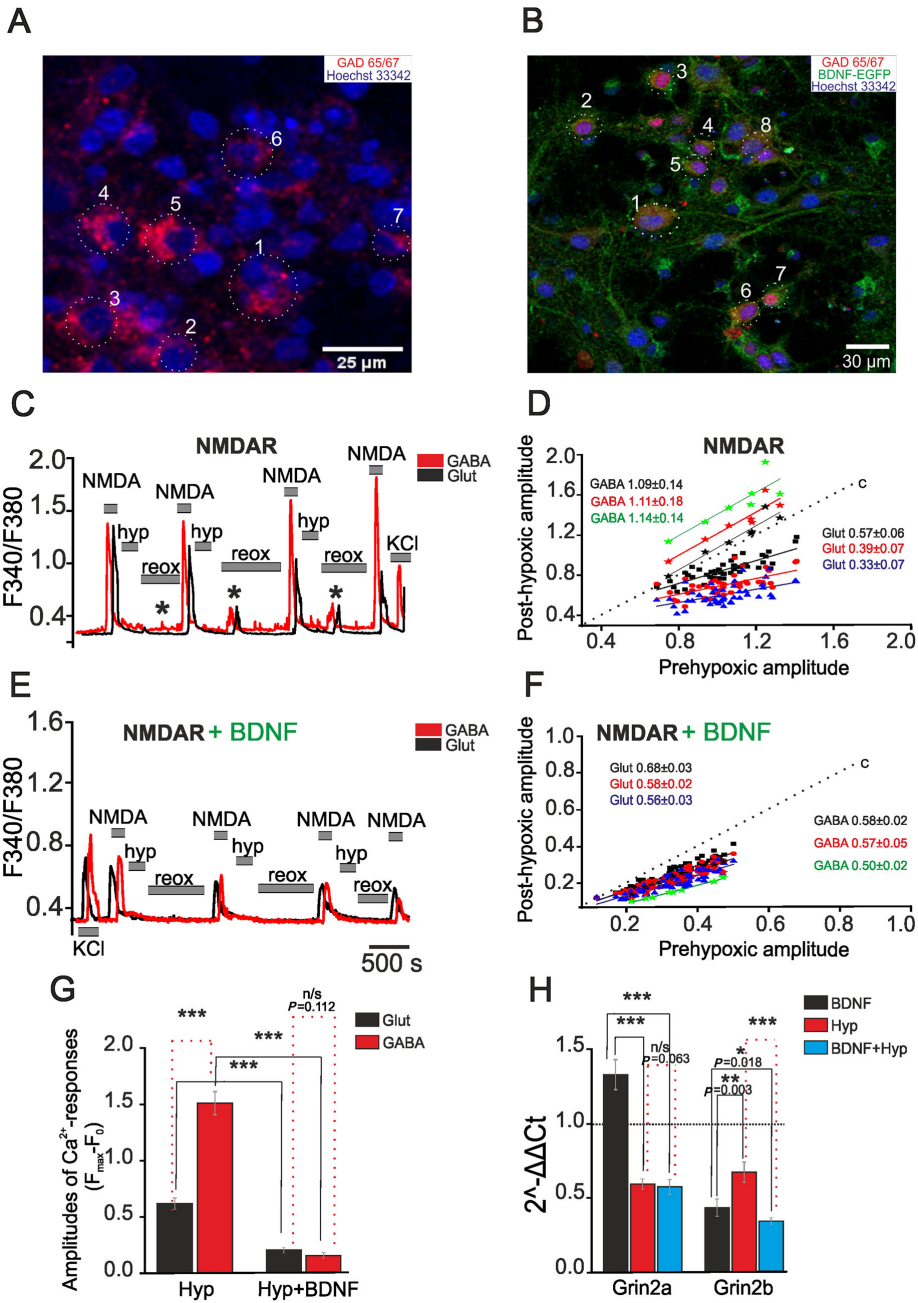
Effects of BDNF overexpression on the amplitude of NMDA-induced Ca^2+^ responses and expression of NMDAR subunits. **A** Identification of GABAergic neurons in a rat hippocampal culture (blue, nuclei stained with Hoechst 33342). Cells 1–7 are GABAergic neurons [averaged Ca^2+^ signal shown as the red trace in **C**, and individual amplitudes in **D** (stars)]. **B** Immunostaining of GABAergic neurons in a culture transduced with (AAV)-Syn-BDNF-EGFP (green). Cells 1–8 are GABAergic neurons [averaged Ca^2+^ signal shown as the red trace in **E**, and individual amplitudes in **F** (stars)]. **C**, **E** Averaged Ca^2+^ responses to NMDA (10 µmol/L in Mg^2+^-free medium) in GABAergic (red trace) and glutamatergic (black trace) neurons from non-transduced cultures (**C**) and those transduced with (AAV)-Syn-BDNF-EGFP (**E**) (hyp, reox: brief episodes of hypoxia and reoxygenation; *spontaneous synchronous Ca^2+^ pulses reflecting post-hypoxic hyperexcitation). **D**, **F** Amplitude of Ca^2+^ responses (arbitrary units) of individual neurons to NMDA after hypoxia/reoxygenation cycles before preconditioning for GABAergic (stars) and glutamatergic neurons from non-transduced cultures (**D**) and those transduced with (AAV)-Syn-BDNF-EGFP (**F**) [dotted lines (c): linear function of data from control cultures (without hypoxia/reoxygenation cycles); black squares, red circles, and green triangles: responses of glutamatergic neurons after the first, second, and third cycles of hypoxia/reoxygenation, respectively; black, red and green stars: responses of GABAergic neurons after the first, second, and third hypoxia/reoxygenation cycles; solid lines in corresponding colors: linear regressions approximating the amplitudes of Ca^2+^ responses for both populations of neurons]. **G** Averaged amplitudes of Ca^2+^ responses of glutamatergic (black) and GABAergic (red) neurons to NMDA (10 µmol/L in Mg^2+^-free medium) after three hypoxia/reoxygenation cycles in non-transduced (Hyp) and transduced cultures (Hyp + BDNF). **H** Effects of neuronal BDNF overexpression on the expression of Grin2a and Grin2b genes in transduced cultures that were not exposed to hypoxia (black) and the transduced cultures 24 h after the preconditioning with short episodes of hypoxia (blue) (red bars (Hyp), levels of Grin2a and Grin2b expression in non-transduced cultures 24 h after preconditioning with brief episodes of hypoxia). The level of expression in non-transduced cultures that were not exposed to hypoxia was set at 1 (dotted line).

GABAergic neurons were identified by immunostaining with antibodies against glutamate GAD65/67, while GAD65/67-negative neurons were glutamatergic (Fig. 1A). It is known that GAD65/67 is expressed exclusively in neurons [29]. The Ca^2+^ responses of GABAergic and glutamatergic neurons to brief applications of NMDA (10 μmol/L in Mg^2+^-free medium), a selective agonist of NMDARs, were found to decrease after hypoxia/reoxygenation cycles (Fig. 1C). In glutamatergic neurons, the slope of the curve approximating the ratio of the amplitude of NMDA-induced responses after a hypoxic episode to the amplitude before hypoxia was 0.57 ± 0.06 after the first episode, 0.39 ± 0.07 after the second, and 0.33 ± 0.07 after the third. This hypoxia-induced decrease of amplitudes is considered to be an expression of HP [11]. On the contrary, the amplitudes of NMDA-induced responses increased in GABAergic neurons after hypoxia-reoxygenation cycles (Fig. 1C), with a slope of 1.09 ± 0.14 after the first, 1.11 ± 0.18 after the second, and 1.14 ± 0.14 after the third cycle (Fig. 1D). These data showed a lack of HP in GABAergic neurons. The shape of the Ca^2+^ responses of glutamatergic neurons to repetitive applications of NMDA did not significantly change in experiments without hypoxia (Fig. S3A) and the slope of regression line was 0.97 ± 0.03 after the second, 0.93 ± 0.05 after the third, and 1.02 ± 0.02 after the fourth NMDA application (Fig. S3B). Similarly, the amplitudes of Ca^2+^ responses of GABAergic neurons also did not change (Fig. S3A), and the corresponding slopes were 0.93 ± 0.08, 0.89 ± 0.16, and 0.91 ± 0.06 (Fig. S3B). However, the amplitudes of Ca^2+^ responses of GABAergic neurons were, on average, higher than those of glutamatergic neurons (Fig. S3I).

BDNF overexpression was found in cultures 2–3 days after transduction with (AAV)-Syn-BDNF-EGFP (Fig. 1B). The amplitude of NMDA-induced Ca^2+^ responses in glutamatergic neurons before hypoxia was lower in cultures with BDNF overexpression than in controls (Fig. 1E). The Ca^2+^ responses of individual glutamatergic neurons both before and after hypoxic episodes was right-shifted along the X-axis (Fig. 1F), compared to the GABAergic neurons (Fig. 1D). The slope of linear regression (Fig. 1F) for glutamatergic neurons was 0.68 ± 0.03 after the first, 0.58 ± 0.02 after the second, and 0.56 ± 0.03 after the third hypoxia-reoxygenation cycle. It should be noted that the amplitudes of NMDA-induced Ca^2+^ responses of GABAergic neurons (Fig. 1E) were lower in cultures with neuronal BDNF overexpression than in controls, indicating the induction of HP. The slope of linear regression for GABAergic neurons was 0.58 ± 0.02 after the first, 0.57 ± 0.05 after the second, and 0.50 ± 0.02 after the third hypoxia/reoxygenation cycle (Fig. 1F). The amplitudes of NMDA-induced Ca^2+^ responses of GABAergic neurons before hypoxia were also lower than in controls. We also compared the average amplitudes of the responses of GABAergic and glutamatergic neurons to NMDA application after three hypoxia/reoxygenation cycles (Fig. 1G). The amplitudes of Ca^2+^ responses of glutamatergic neurons decreased by 64% in cultures with BDNF overexpression compared to non-transduced cultures (Fig. 1G). In turn, the amplitude in GABAergic neurons decreased by 92%. Interestingly, hippocampal neurons generated spontaneous Ca^2+^ pulses during reoxygenation that can be considered post-hypoxic hyperexcitation (Fig. 1C). Such pulses can sometimes turn into irreversible [Ca^2+^]_i_ elevation that leads to damage and death of the most vulnerable populations of neurons, including GABAergic neurons [20]. These Ca^2+^ pulses did not occur in cultures transduced with the (AAV)-Syn-BDNF-EGFP construct. Thus, post-hypoxic hyperexcitation is suppressed by neuronal BDNF overexpression. According to the slope values (Fig. S3D), the amplitudes of the Ca^2+^ responses of GABAergic and glutamatergic neurons induced by repetitive NMDA treatment did not change in the absence of episodes of hypoxia in the transduced cultures with BDNF overexpression (Fig. S3C). Similar to non-transduced cultures, the average amplitudes of the responses of GABAergic neurons in the transduced cultures were higher than those of glutamatergic neurons (Fig. S3I). However, the amplitudes of the responses of GABAergic and glutamatergic neurons were significantly lower (GABAergic, *P *= 0.021; glutamatergic, *P *= 0.012) in the cultures with BDNF overexpression than in non-transduced cultures (Fig. S3I).

Expression of the Grin2a gene, which encodes the NR2A subunit of the NMDAR, was 48% higher in cultures with BDNF overexpression than in controls (Fig. 1H). Meanwhile, the expression of the Grin2b gene, which encodes the NR2B subunit, was 57% lower in the transduced cultures (Fig. 1H).

The expression of Grin2a decreased by 41% and that of Grin2b by 33% in non-transduced cultures 24 h after preconditioning with three episodes of hypoxia (Fig. 1H). In turn, the expression of Grin2a was 43% lower in transduced cultures 24 h after the preconditioning than in cultures without any exposure. However, the difference between the preconditioned transduced and non-transduced cultures was not significant (Fig. 1H). The expression of Grin2b in the transduced cultures decreased by 67% compared to controls (without exposure) after hypoxic preconditioning and by 52% compared to non-transduced cultures after hypoxia (Fig. 1H).

**Fig. 2:**
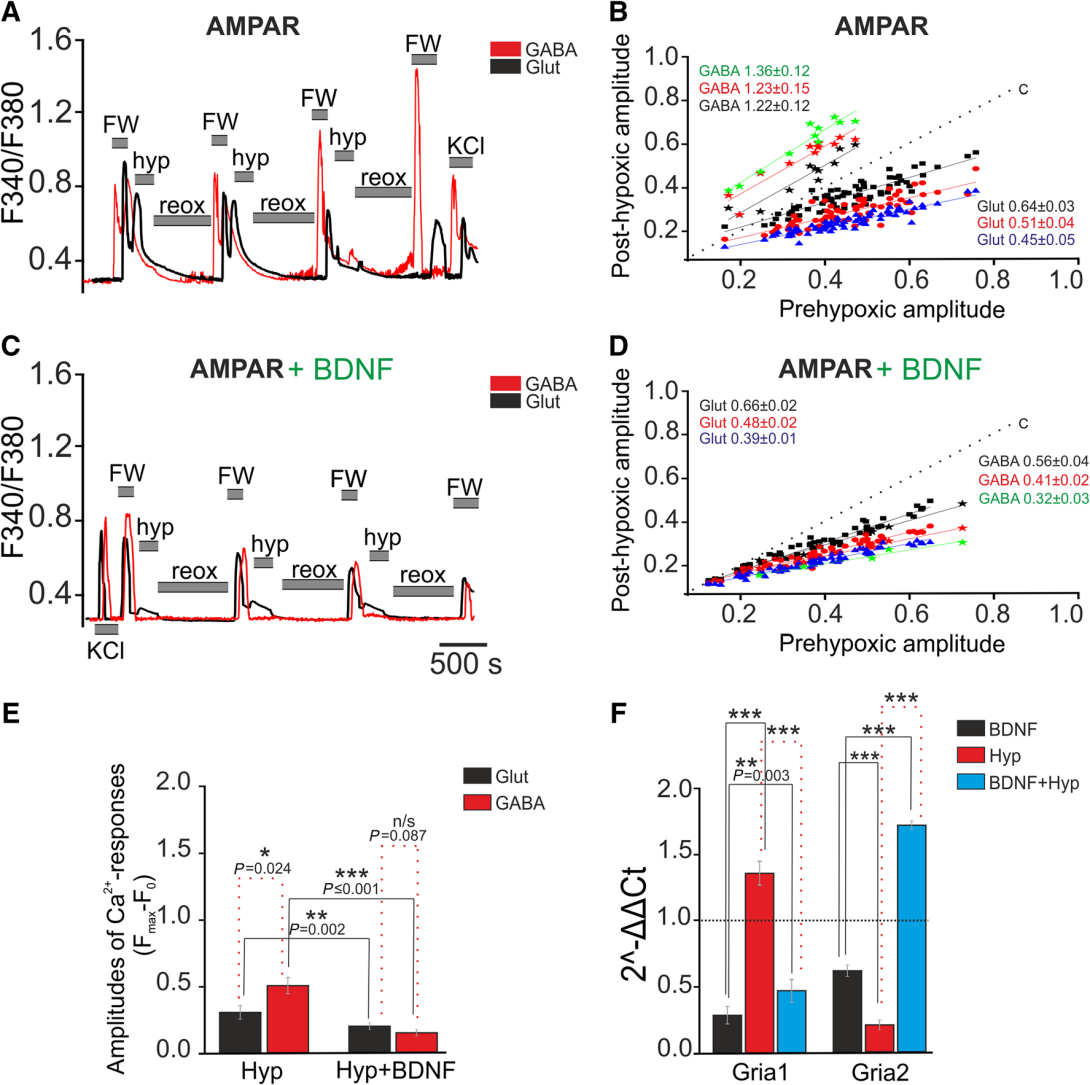
Effects of BDNF overexpression on the amplitude of AMPAR-mediated Ca^2+^ responses and the expression of AMPAR subunits. **A**, **C** Averaged Ca^2+^ responses to FW application (0.3 µmol/L) in GABAergic (red traces) and glutamatergic (black traces) neurons from non-transduced cultures (**A**) and those transduced with (AAV)-Syn-BDNF-EGFP (**C**) (hyp, reox: brief episodes of hypoxia and reoxygenation). **B**, **D** Dependence of amplitudes of Ca^2+^ responses (arbitrary units) to FW after hypoxia/reoxygenation cycles on the amplitudes before preconditioning in GABAergic (stars) and glutamatergic neurons from non-transduced cultures (**B**) and those transduced with (AAV)-Syn-BDNF-EGFP (**D**) (black squares, red circles, and blue triangles: responses of glutamatergic neurons after the first, second, and third cycles of hypoxia/reoxygenation, respectively: black, red, and green stars: responses of GABAergic neurons after the first, second, and third hypoxia/reoxygenation cycles, respectively; color-coded solid lines: corresponding linear regressions; dotted line (c): linear regression for control cultures without hypoxia/reoxygenation). **E** Averaged amplitudes of Ca^2+^ responses of glutamatergic (black) and GABAergic (red) neurons to FW application after three hypoxia/reoxygenation cycles in non-transduced and transduced cultures. **F** Effects of neuronal BDNF overexpression on the expression of Gria1 and Gria2 genes in transduced cultures not exposed to hypoxia (black) and 24 h after preconditioning with short episodes of hypoxia (blue) (red bars: levels of Gria1 and Gria2 expression in non-transduced cultures 24 h after preconditioning with brief episodes of hypoxia). The level of expression in non-transduced cultures not exposed to hypoxia was set at 1 (dotted line). ***P ≤ 0.001.

Repetitive brief application of 5-fluorowillardiine (FW), a selective AMPAR agonist (0.3 µmol/L), induced Ca^2+^ responses in GABAergic and glutamatergic neurons. These responses were similar to NMDA-induced responses, and the slopes of the linear regressions were close to 1 in cultures without hypoxia (Fig. S3E, F). Nevertheless, the amplitudes of FW-induced Ca^2+^ responses of GABAergic neurons were significantly higher than those of glutamatergic neurons (*P *≤ 0.001; Fig. S3J). Brief episodes of hypoxia decreased the amplitude of AMPAR-mediated Ca^2+^ responses in glutamatergic neurons (Fig. 2A). The slope of the linear regression decreased gradually (Fig. 2B) and was 0.64 ± 0.03 after the first, 0.51 ± 0.04 after the second, and 0.45 ± 0.05 after the third hypoxia/reoxygenation cycle. In contrast, the amplitudes of FW-induced Ca^2+^ responses of GABAergic neurons increased after the hypoxia/reoxygenation cycles (Fig. 2A); the slope of regression was 1.36 ± 0.12 after the first, 1.23 ± 0.15 after the second, and 1.22 ± 0.12 after the third cycle (Fig. 2B). Therefore, the activity of AMPARs of GABAergic neurons is not affected by brief episodes of hypoxia.

However, the amplitudes of the FW-induced Ca^2+^ responses of GABAergic neurons after episodes of hypoxia/reoxygenation decreased in cell cultures transduced with (AAV)-Syn-BDNF-EGFP (Fig. 2C). The slope of the linear regression in this case was 0.56 ± 0.04 after the first, 0.41 ± 0.02 after the second, and 0.32 ± 0.03 after the third hypoxia/reoxygenation cycle (Fig. 2D). Interestingly, neuronal BDNF overexpression dramatically enhanced the preconditioning effect of hypoxia in glutamatergic neurons. The amplitudes of FW-induced Ca^2+^ responses of these neurons substantially decreased (Fig. 2C) in (AAV)-Syn-BDNF-EGFP-transduced cultures after hypoxia-reoxygenation cycles compared to those in non-transduced cultures. The slope values were significantly lower than those in non-transduced cultures (0.66 ± 0.02 after the first, 0.48 ± 0.02 after the second, and 0.39 ± 0.01 after the third cycle) (Fig. 2D).

The averaged amplitudes of FW-induced Ca^2+^ responses of glutamatergic neurons after three hypoxia/reoxygenation cycles were 23% lower in cultures with neuronal BDNF overexpression than those in non-transduced cultures (*P *= 0.002; Fig. 2E). In addition, the averaged amplitudes of GABAergic neurons decreased by 62% (*P *≤ 0.001; Fig. 2E).

The amplitudes of repetitive FW-induced Ca^2+^ responses of GABAergic and glutamatergic neurons did not change in cultures with BDNF overexpression (Fig. S3G, H). Interestingly, BDNF overexpression promoted a decrease in the amplitudes of FW-induced responses only in GABAergic neurons (Fig. S3J), while the changes in the amplitudes of glutamatergic neurons were insignificant (*P *= 0.725). However, the amplitudes of the responses of GABAergic neurons in cultures with BDNF overexpression were higher than those of glutamatergic neurons (Fig. S3J; *P *= 0.017).

The basal expression of the Gria1 and Gria2 genes that encode the GluA1 and GluA2 subunits of AMPARs decreased by 71.3% and 38.5% respectively in (AAV)-Syn-BDNF-EGFP-transduced cultures compared to control cultures not exposed to hypoxia (Fig. 2F). Gria1 expression increased by 35% in non-transduced cell cultures 24 h after preconditioning with hypoxia/reoxygenation episodes, while Gria2 expression decreased by 80%; in contrast, Gria1 expression decreased by 53%, while Gria2 expression increased by 71% in the transduced cultures 24 h after preconditioning (Fig. 2F).

We noted that the amplitudes of the NMDAR- and AMPAR-mediated Ca^2+^ responses of GABAergic neurons were always higher than those of glutamatergic neurons. BDNF overexpression promoted the development of HP in GABAergic neurons, thus decreasing the amplitudes of NMDAR- and AMPAR-mediated Ca^2+^ responses. The amplitudes of repetitive NMDAR- and AMPAR-mediated responses did not change in GABAergic or glutamatergic neurons without episodes of hypoxia/reoxygenation. BDNF overexpression promoted the decrease in the amplitudes of NMDAR-mediated responses of both GABAergic and glutamatergic neurons, while AMPAR-mediated responses decreased only in GABAergic neurons. BDNF overexpression promoted the development of HP in GABAergic neurons. This phenomenon appeared as a decrease in Ca^2+^ response amplitudes upon activation of NMDARs and AMPARs. In addition, the preconditioning effect of hypoxia was enhanced in glutamatergic neurons from transduced cultures. The amplitudes of Ca^2+^ responses to the activation of AMPARs and NMDARs before episodes of hypoxia were lower in both GABAergic and glutamatergic neurons from cultures with BDNF overexpression. This effect can be explained by BDNF-mediated changes in the expression of the genes encoding the subunits that regulate the Ca^2+^ permeability of the receptors. Grin2a and Grin2b expression was lower 24 h after repetitive episodes of hypoxia/reoxygenation in the (AAV)-Syn-BDNF-EGFP-transduced cultures than in control cultures not exposed to hypoxia. However, we found that Gria1 expression was increased 24 h after hypoxia/reoxygenation cycles in controls, while Gria2 expression was decreased. In contrast, the decreased Gria1 and increased Gria2 expression occurred 24 h after exposure to brief episodes of hypoxia in cultures with BDNF overexpression in neurons. This finding indicates that the decreased Ca^2+^ permeability of AMPARs is due to the weak synthesis of GluA2 subunits that are known to regulate Ca^2+^ conductivity.

### BDNF Overexpression Enhances the Protective Effect of Hypoxic Preconditioning Against OGD-Induced Cell Death

**Fig. 3:**
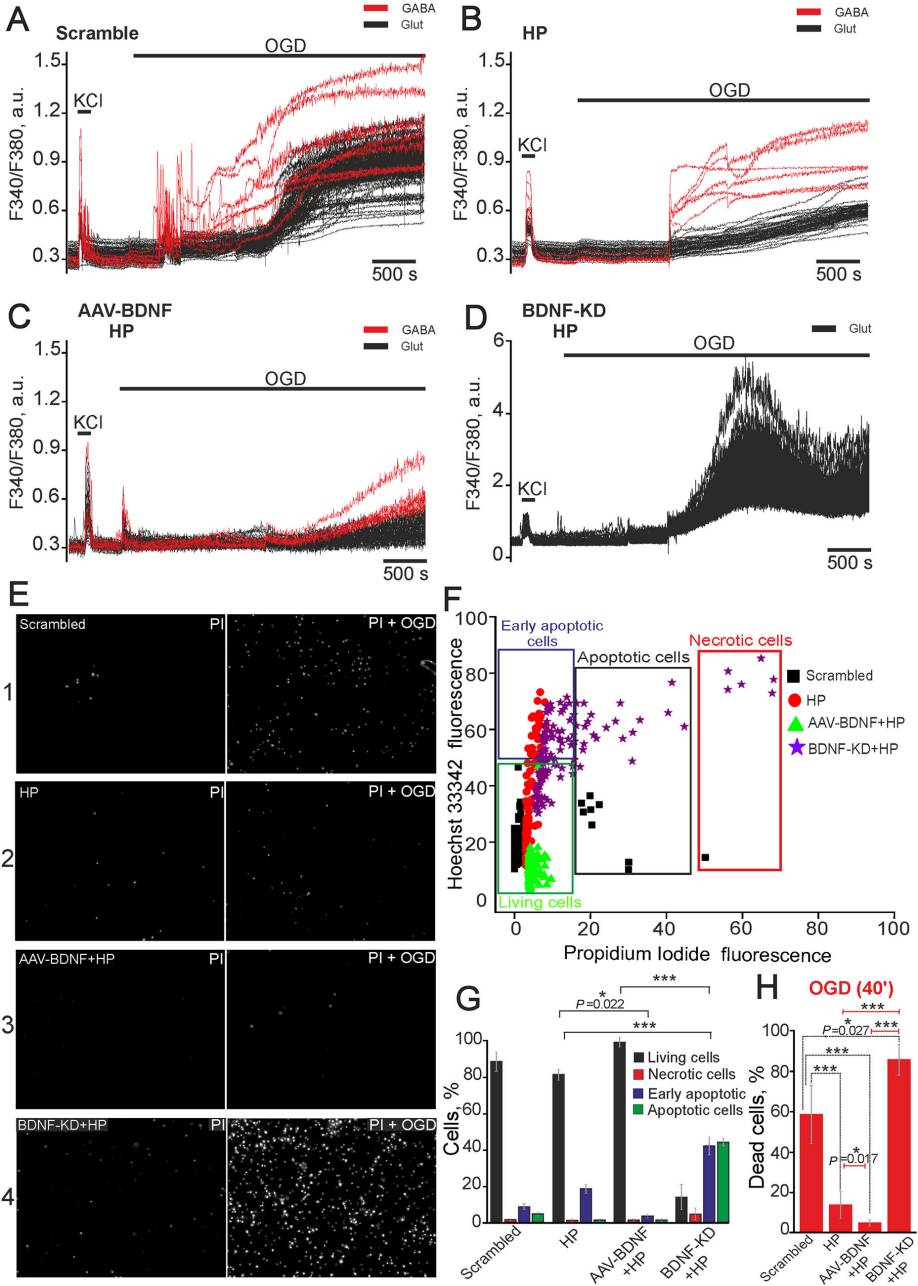
Protective action of brief periods of hypoxic preconditioning and BDNF overexpression against OGD-induced Ca^2+^ overload and cell death. **A**–**D** OGD-induced Ca^2+^ responses of glutamatergic (black traces) and GABAergic neurons (red traces) in cultures: (**A**) transfected with an siRNA whose sequence differed from that of the siRNA against BDNF (Scrambled, negative control); (**B**) preconditioned with brief episodes of hypoxia-reoxygenation (HP); (**C**) transduced with (AAV)-Syn-BDNF-EGFP and preconditioned with HP; (**D**) transfected with siRNA targeting the BDNF gene and preconditioned with HP. **E** Effects of BDNF overexpression on cell survival in preconditioned hippocampal cultures after 40-min OGD. Representative images of PI-stained cultures (white dots) before (left panels) and after OGD (right panels). 1: cultures transfected with an siRNA whose sequence differed from that of the siRNA against BDNF; 2: cultures preconditioned with HP; 3: cultures transduced with (AAV)-Syn-BDNF-EGFP and preconditioned with HP; 4: cultures transfected with siRNA targeting the BDNF gene preconditioned with HP. **F** Effects of BDNF overexpression and hypoxic preconditioning on the induction of apoptosis and necrosis in cultures before OGD. The cytogram shows the viability of hippocampal cells (X-axis, intensity of PI fluorescence; Y-axis, intensity of Hoechst 33342 fluorescence; cells were stained with probes 24 h after preconditioning). **G** Percentages of living (black), necrotic (red), and early (violet) and late (green) apoptotic cells in different experimental groups before OGD (two-way ANOVA followed by *post-hoc* Tukey-Kramer test; comparisons between experimental groups but not between individual columns; all differences were significant: ***P ≤ 0.001, HP *vs* Scrambled, AAV-BDNF + HP *vs* Scrambled, and BDNF-KD + HP *vs* Scrambled; *P = 0.022, HP *vs* BDNF-KD + HP; ***P ≤ 0.001, HP *vs* BDNF-KD + HP, AAV-BDNF + HP *vs* BDNF-KD + HP). **H** Percentages of dead cells after 40-min OGD in different experimental groups as in **E** [one-way ANOVA followed by *post-hoc* Tukey-Kramer test; ***P ≤ 0.001, HP *vs* Scrambled, AAV-BDNF + HP *vs* Scrambled AAV-BDNF, AAV-BDNF + HP *vs* BDNF-KD + HP, and HP *vs* BDNF-KD + HP; *P = 0.027, BDNF-KD + HP *vs* Scrambled and HP *vs* AAV-BDNF + HP; *n* = 3 for **F** and **G]**. After preconditioning, the cultures were returned to a CO_2_ incubator for 24 h.

To investigate the contribution of BDNF to the development of the HP effect and the activation of the protective signaling cascades in GABAergic neurons, we knocked down BDNF in cultures using siRNA. The OGD experiments were performed 48 h after transfection (Fig. 3). Cultures transfected with an siRNA whose sequence differed from that of the siRNA against BDNF (Scrambled) were used as controls (Fig. 3A). We found that the process of transfection did not itself induce cell death because the percentage of early apoptotic cells in the scrambled group was similar to that in control cultures (without exposure; Fig. 7B) and accounted for ~ 10% of cells (Fig. 3F, G); only individual necrotic cells were observed (Fig. 3E). Necrotic, as well as early and late apoptotic cells were identified using PI and Hoechst 33342. A biphasic increase in [Ca^2+^]_i_ (Fig. 3A) was detected in glutamatergic and GABAergic neurons during 40-min OGD in the scrambled group. GABAergic neurons were characterized by higher amplitudes of Ca^2+^ signals during the first phase of the response, which was immediately followed by the second phase (irreversible [Ca^2+^]_i_ increase). In contrast, a small lag period between the first and second phases was found in glutamatergic neurons (Fig. 3A). The duration of this lag varied from culture to culture and may be dependent on the activity of Ca^2+^-transporting systems in neurons. The death of 60% ± 18% cells occurred after 40 min of OGD (Fig. 3E, H).

The cultures preconditioned with brief episodes of hypoxia following the experimental protocol presented in Fig. 1 were returned to a CO_2_ incubator for 24 h, after which they were used in OGD experiments. We detected only individual necrotic cells in the preconditioned cultures (Fig. 3E), while the proportion of early apoptotic cells was 17% ± 4% (Fig. 3F, G). In turn, the proportion of late apoptotic cells was ~ 1%—lower than in the scrambled group. Therefore, it can be assumed that HP induces apoptosis in a small group of cells on the one hand, and promotes the appearance of a lag phase that may suppress the development of apoptosis on the other hand. The first phase of the OGD-induced Ca^2+^ response was virtually suppressed in the preconditioned cultures, and the amplitudes of Ca^2+^ responses during the second phase were 60% lower (Fig. 3B) than in the scrambled group (Fig. 3A). A prolonged lag period preceding the OGD-induced Ca^2+^ responses appeared in GABAergic neurons from preconditioned cultures (Fig. 3B). However, the preconditioning did not abolish the irreversible [Ca^2+^]_i_ increase. HP promoted a decrease in OGD-induced cell death to 15% ± 6% (Fig. 3E, H).

Preconditioning of cultures with BDNF overexpression caused a decrease in the proportion of early apoptotic cells to 1% (Fig. 3F, G). Moreover, necrotic cells were not detected in this group before OGD (Fig. 3E). The second phase of the OGD-induced Ca^2+^ response was completely suppressed in glutamatergic neurons from these cultures, while the first phase was not affected, but in GABAergic neurons, the amplitudes of the responses were 59% lower than in the scrambled group (Fig. 3C). Similar to glutamatergic neurons, the first phase was also unaffected by preconditioning. The proportion of dead cells after OGD decreased to 5% ± 3% (Fig. 3E, H).

As noted above, to suppress BDNF expression, the cells were transfected with siRNA against BDNF. The efficiency of transfection and BDNF knockdown were determined by immunostaining and RT-PCR assay 48 h later. The cells were stained with antibodies against BDNF and NeuN (Fig. S2A, B) and the fluorescence of anti-BDNF antibodies was detected in most neurons and some NeuN-negative cells from the negative control group (scrambled; Fig. S2A). In contrast, fluorescence of anti-BDNF antibodies was not observed in any cells from the siRNA-transfected cultures (Fig. S2B, C). RT-PCR assays revealed that expression of the gene encoding BDNF was almost completely suppressed in cultures with BDNF knockdown (Fig. S2D), while the expression in the scrambled group was similar to controls (without exposure). Thus, all subsequent experiments were performed using cultures with confirmed BDNF knockdown.

Preconditioning of the transfected cultures with episodes of hypoxia led to an increase of necrotic cells to 8% ± 3% (Fig. 3G, E), while the proportion of apoptotic cells was 85%, including 40% ± 12% early and 43% ± 7% late apoptotic cells (Fig. 3F, G). Two phases of the OGD-induced Ca^2+^ response occurred in glutamatergic neurons (Fig. 3D) in cultures with BDNF knockdown, and the amplitude of the Ca^2+^ signal during the second phase was 4 times that of the other experimental groups. Immunostaining of the transfected cultures with anti-GAD65/67 antibodies revealed the absence of GABAergic neurons, indicating their death during preconditioning. The percentage of dead cells after OGD reached 87% ± 13% (Fig. 3H). The transfection procedure did not cause these effects of BDNF knockdown on GABAergic neurons because these neurons were detected after OGD in the scrambled group and the basal percentage of dead cells was similar to controls (Lipofectamine RNAiMAX and siRNA without additional exposure) (Fig. 7).

**Fig. 4:**
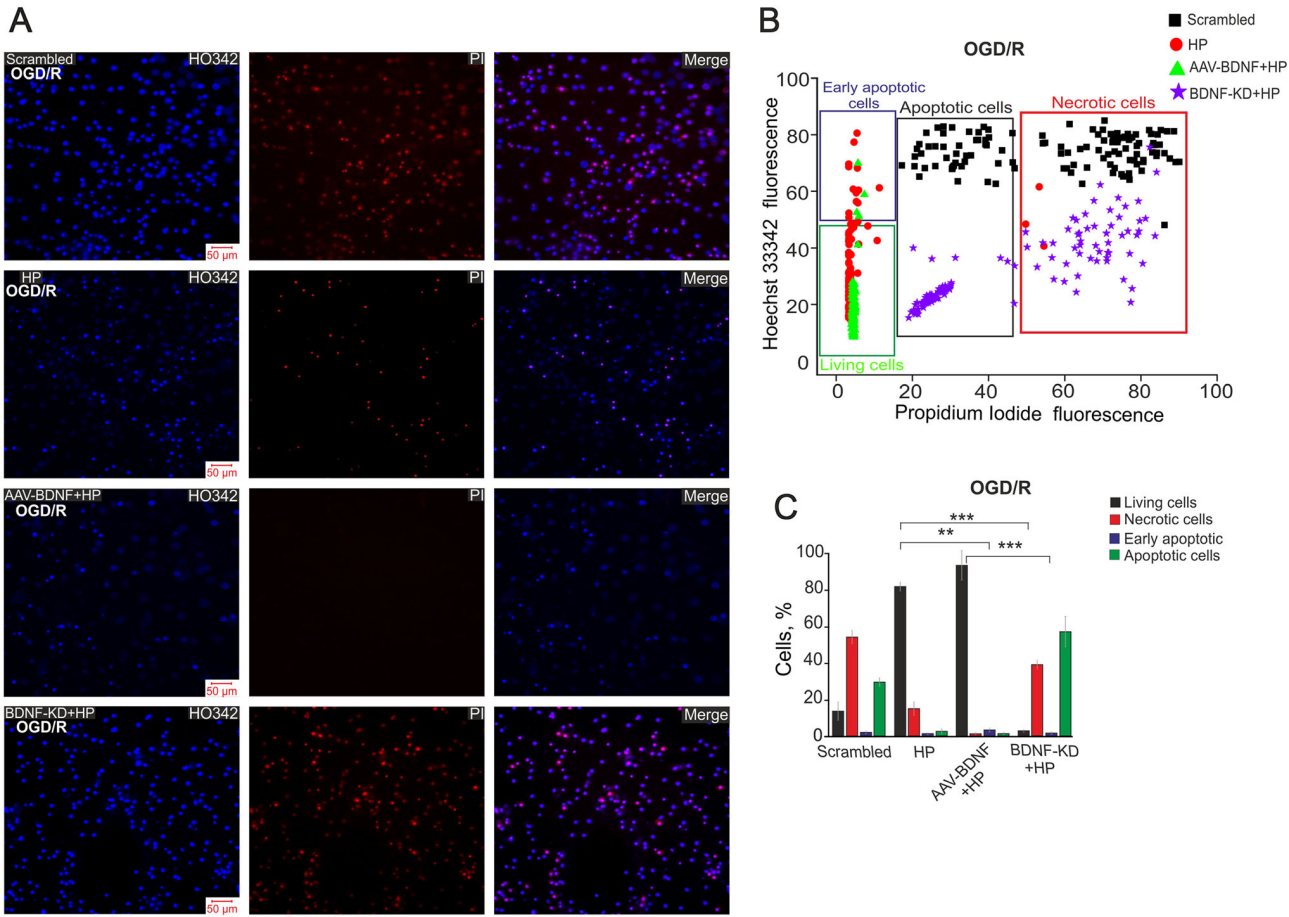
Protective effect of hypoxic preconditioning and BDNF overexpression 24 h after OGD. **A** Double staining of cells with Hoechst 33342 (HO342) and propidium iodide (PI) and merged images (Merge) 24 h after 40-min OGD. **B** Cytogram of the viability of hippocampal cells (X-axis, intensity of PI fluorescence; Y-axis, intensity of Hoechst 33342 fluorescence; cells were stained with probes 24 h after HP). **C** Effects of HP, BDNF, and its knockdown on the induction of necrosis and apoptosis 24 h after OGD. Percentages of living cells (black) and cells in which early apoptosis (violet), apoptosis (green), and necrosis (red) were detected. Cultures were returned to a CO_2_ incubator for 24 h after HP, then used in OGD experiments (40-min OGD) and returned to the CO_2_ incubator for 24 h, after which, they were stained with HO342 and PI. (***P ≤ 0.001, HP *vs* Scrambled, AAV-BDNF + HP *vs* Scrambled, and BDNF-KD + HP *vs* Scrambled; **P = 0.008, HP *vs* AAV-BDNF + HP; ***P ≤ 0.001, HP *vs* BDNF-KD + HP and AAV-BDNF + HP *vs* BDNF-KD + HP).

To investigate the delayed effects of BDNF overexpression and HP under OGD, the preconditioned cultures were exposed to 40-min OGD and returned to a CO_2_ incubator for 24 h. Next, the cells were stained with PI and Hoechst 33342 (HO342). This model can be considered as more toxic because replacement of the ischemic medium leads to reoxygenation that promotes apoptosis and necrosis after OGD [30]. The increased fluorescence intensity of HO342 and PI in the scrambled group (without preconditioning) indicated the activation of apoptosis and necrosis 24 h after OGD (Fig. 4A). The percentage of late-apoptotic cells was 32% ± 7% and of necrotic cells 57% ± 9% (Fig. 4B, C), while early-apoptotic cells were virtually absent in this group. The cells preconditioned with HP mainly survived 24 h after OGD (82% ± 6%, Fig. 4A). The percentage of necrotic cells was 17% ± 8%, and only individual apoptotic cells were detected (Fig. 4B, C).

Necrotic cells were not detected in the preconditioned cell cultures with BDNF overexpression (Fig. 4A), while the percentage of apoptotic cells was similar to that in preconditioned cultures (Fig. 4B, C). Thus, BDNF overexpression enhanced the protective effect of HP, suppressing necrosis and increasing the percentage of living cells to 90% ± 10% (Fig. 4C).

We found massive cell death after OGD in the preconditioned cultures with BDNF knockdown (Fig. 4A). According to the fluorescence intensity of HO342 and PI, the percentage of late apoptotic cells was 62% ± 18%, while that of necrotic cells was 41% ± 7% (Fig. 4C).

Thus, preconditioning of hippocampal cell cultures with brief episodes of hypoxia promoted the decrease of OGD-induced Ca^2+^ responses in glutamatergic neurons, and the percentages of necrotic cells immediately after OGD, as well as 24 h later, decreased in this case. The OGD-induced responses of glutamatergic as well as GABAergic neurons were significantly reduced in the transduced cultures after HP. BDNF enhanced the neuroprotective effect of HP, promoting cell survival after OGD and completely suppressing cell death. Experiments with BDNF knockdown confirmed this conclusion. Massive apoptotic and necrotic cell death occurred in the cultures with suppressed BDNF expression and the OGD-induced Ca^2+^ influx was more intense. Interestingly, GABAergic neurons were not detected by immunostaining in cultures with BDNF knockdown. Most of the cells were identified as late apoptotic or necrotic 24 h after 40-min OGD in the cultures with knockdown. Thus, BDNF can be considered as a pivotal neurotrophic factor protecting hippocampal cells, especially GABAergic neurons, against OGD-induced damage. HP had no neuroprotective effect in cultures with BDNF knockdown, and the loss of GABAergic neurons possibly occurred during preconditioning.

### BDNF Overexpression Alters the Basal and Hypoxia-induced Expression of Genes Involved in the Development of Hypoxic Preconditioning

**Fig. 5:**
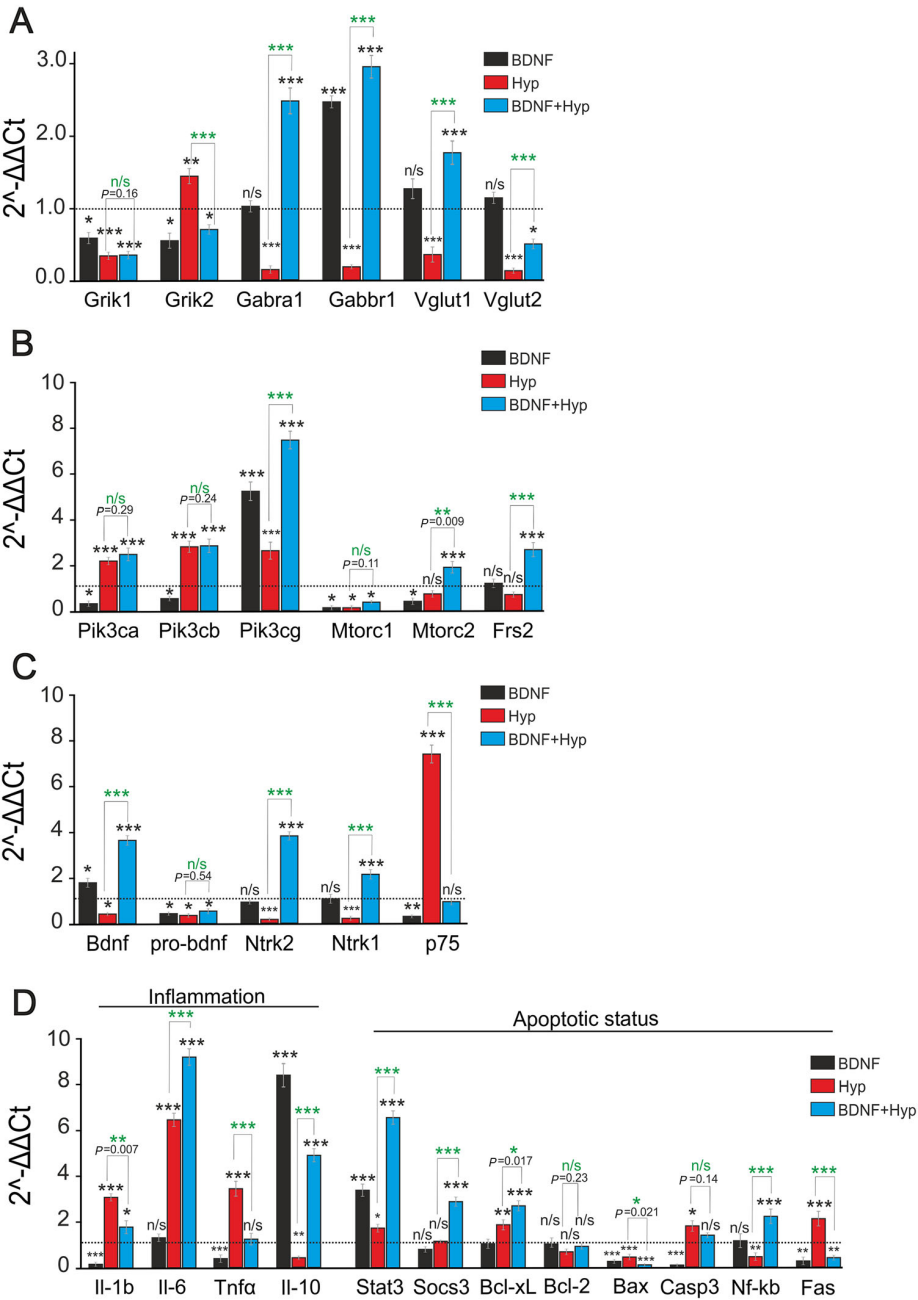
Effects of BDNF overexpression in neurons on the expression of genes in controls and after brief episodes of hypoxia/reoxygenation. **A**–**C** Expression levels of: (**A**) genes encoding subunits of kainate receptors (Grik1 and Grik2), GABA receptors (Gabra1 and Gabbr1), and genes encoding vesicular glutamate transporters (Vglut1 and Vglut2); (**B**) genes encoding subunits of PI3-kinase (Pik3ca, Pik3cb, and Pik3cg), genes of the target of rapamycin (Mtorc1 and Mtorc2), and the gene of fibroblast growth factor receptor substrate 2 (Frs2); (**C**) genes encoding BDNF and its precursor, pro-BDNF, along with those encoding the receptors of pro-BDNF and BDNF (Ntrk1, Ntrk2, and p75). **D** Expression levels of genes that regulate inflammation and apoptosis [dashed line level of gene expression in controls (non-transduced cell cultures without hypoxia) was set at 1]. ***P ≤ 0.001 (green asterisks: significance of differences between non-transduced groups with and without BDNF).

Real-time PCR analysis showed that BDNF overexpression in hippocampal neurons led to a decrease in Grik1 expression by 42% (*P *= 0.014) and in Grik2 by 46% (*P *= 0.013) compared to controls (Fig. 5). These genes encode the GluK1 and GluK2 subunits of kainate receptors. Grik1 expression was reduced by 67% (*P *≤ 0.001) 24 h after HP in non-transduced cultures, while Grik2 expression increased by 43% (*P *= 0.0016). Grik1 expression was reduced by 65% (*P *≤ 0.001) and Grik2 expression by 30% (*P *= 0.021) in transduced cultures after HP.

Interestingly, expression of the Gabra1 and Gabbr1 genes, which encode the α1 subunit of GABA_A_ and the β1 subunit of GABA_B_ receptors, decreased by 86% (*P *≤ 0.001) and 81% (*P *≤ 0.001), respectively, 24 h after hypoxia/reoxygenation in non-transduced cultures (Fig. 5A). It can be concluded that the decreased expression of subunits of critical inhibitory receptors leads to attenuation of the inhibition of neuronal networks. However, the expression of these genes dramatically increased 24 h after hypoxia/reoxygenation in the cultures with BDNF overexpression; Gabra1 expression increased by 146% (*P *≤ 0.001) and Gabbr1 expression by 194% (*P *≤ 0.001). The basal Gabra1 expression did not change in the transduced cell cultures, but Gabbr1 expression increased by 147% (*P *≤ 0.001). BDNF overexpression in neurons also changed the expression of the Vglut1 and Vglut2 genes that encode vesicular glutamate transporter 1 and 2. Their expression increased slightly (*P *= 0.27) in transduced cultures without exposure to HP (Fig. 5A). However, the expression levels of these genes decreased by 65% (Vglut1, ***P ≤ 0.001) and 87% (Vglut2, ***P ≤ 0.001) 24 h after HP in cultures without overexpression. Vglut1 expression increased by 76% (*P* ≤ 0.001) in transduced cells after HP. The expression of this gene was higher than the basal expression in transduced cultures without hypoxia. In contrast, Vglut2 expression after hypoxia was 50% lower than control (*P *= 0.011). We also compared the expression of all these genes after HP in transduced and non-transduced cultures (Fig. 5A) and found a significant decrease of Grik2 expression in the transduced cultures (*P *≤ 0.001). In turn, the expression of Gabra1 (*P *≤ 0.001), Gabbr1 (*P *≤ 0.001), Vglut1 (*P *≤ 0.001), and Vglut2 (*P *≤ 0.001) increased, which may be considered a protective effect.

BDNF overexpression led to decreases in the basal expression of the Pik3ca gene by 53% (*P *= 0.014) and the Pik3cb gene by 34% (*P *= 0.026), while Pik3cg expression increased by 428% (*P *≤ 0.001) (Fig. 5B). The expression of these genes in controls increased by 129% for Pik3ca (*P *≤ 0.001), 143% for Pik3cb (*P *≤ 0.001), and 166% for Pik3cg (*P *≤ 0.001) after hypoxic episodes. Pik3ca and Pik3cb expression in the transduced cultures did not change after hypoxia compared to non-transduced cultures (Fig. 5), while the increases were significant compared with controls (*P *≤ 0.001). However, Pik3cg expression increased by 648% (*P *≤ 0.001), substantially exceeding the basal expression in the transduced cultures and the post-hypoxic level in the non-transduced cultures.

Mtorc1 expression decreased by 88% (*P *= 0.012) in non-transduced cultures after HP and in transduced cultures without hypoxia. A lower decrease in Mtorc1 expression by 74% (*P *= 0.022) was found 24 h after HP in cultures with BDNF overexpression. Mtorc2 expression increased by 197% (*P *≤ 0.001) in transduced cell cultures 24 h after hypoxia. In turn, Mtorc2 expression decreased by 55% (*P *= 0.021) in the BDNF group. A decrease was also found in the non-transduced group (24%), but this was not significant (*P *= 0.086). A significant increase of Frs2 expression was found in the BDNF-transduced cell cultures after hypoxia (197%, *P *≤ 0.001; Fig. 5B). However, the changes in Frs2 expression were not significant in the BDNF (*P *= 0.044) and non-transduced (*P *= 0.049) groups. We found significant increases in the expression of Pik3cg (250%, ***P ≤ 0.001), Mtorc2 (121%, **P = 0.009), and Frs (254%, ***P ≤ 0.001) after HP in transduced compared to non-transduced cultures (Fig. 5B).

It is known that BDNF and pro-BDNF activate different cascades with opposite physiological effects [31]. BDNF overexpression in neurons increased basal Bdnf expression by 90% (*P *= 0.021). Nevertheless, pro-BDNF expression decreased by 56% (*P *= 0.006) and that of p75 by 68% (*P *= 0.008) (Fig. 5C). Basal expression of the genes encoding TrkA (Ntrk1) and TrkB (Ntrk2) receptors changed in the range of 10%–12% (*P* = 0.77) in the transduced cultures without hypoxia compared to controls. The expression of Bdnf, pro-BDNF, Ntrk1, and Ntrk2 decreased by 50% (*P *= 0.015), 67% (*P *= 0.015), 75% (*P *≤ 0.001), and 89% (*P *≤ 0.001), respectively, in the non-transduced cultures 24 h after HP. In contrast, p75 expression increased by 646% (*P *≤ 0.001) (Fig. 5C). In turn, Bdnf, Ntrk1, and Ntrk2 expression increased by 270% (*P *≤ 0.001), 115% (*P *≤ 0.001), and 273% (*P *≤ 0.001), respectively, after hypoxia in cultures with BDNF overexpression in neurons. Pro-BDNF expression fell by 50% (*P *= 0.014) in this group, but p75 expression did not differ from its expression in control cultures without hypoxia (*P *= 0.79). In the transduced cultures (Fig. 5C), hypoxic episodes enhanced the expression of the genes encoding BDNF (*P *≤ 0.001) and its receptors (Ntrk1, *P *≤ 0.001; Ntrk2, *P *≤ 0.001) compared to non-transduced cultures. However, expression of the p75 gene was substantially reduced in transduced compared to non-transduced cultures (*P *≤ 0.001).

The basal expression of IL-1β decreased by 85% (*P *≤ 0.001) and TNFα by 47% (*P *= 0.003) in transduced cultures (Fig. 5D), while the difference in IL-6 expression was not significant (*P *= 0.28). IL-10 expression increased by 799% (*P *≤ 0.001) at the same time. IL-1β increased by 218% (*P *≤ 0.001), IL-6 by 596% (*P *≤ 0.001), and TNFα by 265% (*P *≤ 0.001) in non-transduced cultures 24 h after hypoxia, whereas IL-10 expression fell by 54% (*P *= 0.008). IL-1β expression increased by 98% (*P *= 0.022) after hypoxic episodes in cultures with BDNF overexpression. In addition, IL-6 expression increased by 885% (*P *≤ 0.001) and IL-10 by 415% (*P *≤ 0.001). The level of TNFα expression was the same as that in control cultures without hypoxia (*P *= 0.69). Comparing (AAV)-Syn-BDNF-EGFP-transduced and non-transduced cultures, we found that the expression of genes encoding anti-inflammatory cytokines increased after HP (IL-10 by 1043%, *P *≤ 0.001; IL-6 by 53%, *P *= 0.007) in transduced compared to non-transduced cultures (Fig. 5D). In contrast, the expression of genes encoding pro-inflammatory cytokines was reduced (IL-1β by 55%, *P *≤ 0.001; TNFα by 124%, *P *≤ 0.001) in cultures with BDNF overexpression compared to non-transduced cultures.

Stat3 expression was 254% higher than controls (*P *≤ 0.001) in cultures with BDNF overexpression in neurons. Furthermore, expression of the pro-apoptotic genes Bax, Casp3, and Fas was lower by 74% (*P *≤ 0.001), 98% (*P *≤ 0.001), and 77% (*P *= 0.004) than controls. Stat3 and Bcl-xL expression increased by 85% (*P *= 0.019) and 100% (*P *= 0.005) after hypoxic episodes in non-transduced cell cultures, while the expression of the pro-apoptotic genes Bax and NF-κB decreased by 63% (*P *≤ 0.001) and 58% (*P *= 0.004), thus suppressing apoptosis. However, the expression of the pro-apoptotic genes Casp3 and Fas in the non-transduced group increased by 87% (*P *= 0.018) and 123% (*P *≤ 0.001), contributing to apoptosis activation (Fig. 5D). Enhanced expression of anti-apoptotic genes was found 24 h after HP in cultures with BDNF overexpression. Stat3, Socs3, and Bcl-xL expression increased by 589% (*P *≤ 0.001), 203% (*P *≤ 0.001), and 187% (*P *≤ 0.001), respectively. In contrast, the expression of most pro-apoptotic genes was reduced, but the expression of NF-κB increased by 174% (*P *≤ 0.001). Bax expression fell by 93% (*P *≤ 0.001) and Fas by 56% (*P *= 0.006), while the increase of Casp3 expression was not significant (*P *= 0.24) (Fig. 5D). Interestingly, although the expression of many pro- and anti-apoptotic genes significantly changed with hypoxia or BDNF expression and hypoxia, the changes of Bcl-2 and NF-κB expression in the BDNF group without hypoxia were not significant. We also compared the expression of these genes in (AAV)-Syn-BDNF-EGFP-transduced and non-transduced cultures after HP. In the transduced cultures, hypoxia increased the expression of Stat3 by 277% (*P *≤ 0.001), Socs3 by 210% (*P *≤ 0.001), Bcl-xL by 36% (*P *= 0.017), and NF-κB by 287% (*P *≤ 0.001) compared to non-transduced cultures, while the expression of Bax and Fas decreased by 67% (*P *= 0.021) and 312% (*P *≤ 0.001). The expression of the other genes regulating apoptosis did not significantly differ between the non-transduced groups with and without BDNF.

Thus, the expression of most genes was decreased 24 h after HP in non-transduced cultures compared to control cultures without episodes of hypoxia. However, only the decreased Grik1, Bax, and NF-κB expression can be considered neuroprotective. The expression of 8 of the 41 studied genes was increased. Only the increased expression of PI3K, Bcl-xL, and IL-6 can be considered a positive effect, while that of p75, IL-1β, TNFα, and Fas can promote apoptosis and inflammation. The expression of 20 out of the 33 studied genes was increased 24 h after hypoxia in (AAV)-Syn-BDNF-EGFP-transduced cultures compared to the basal expression in these cultures. It should be noted that 16 of these genes encode proteins involved in neuroprotection. Vglut2 expression decreased, while Vglut1 expression increased, indicating a possible compensatory effect. The suppressed Fas expression can be considered a protective effect of BDNF overexpression. So, BDNF overexpression in neurons not only enhances the basal expression of the protective genes and suppresses the expression of pro-apoptotic or pro-inflammatory genes, but also strengthens the protective effects of HP. Moreover, hypoxia leads to a decrease in the expression of a smaller number of protective genes.

### Vesicular Release of BDNF Mediates Its Neuroprotective Action Under OGD

**Fig. 6:**
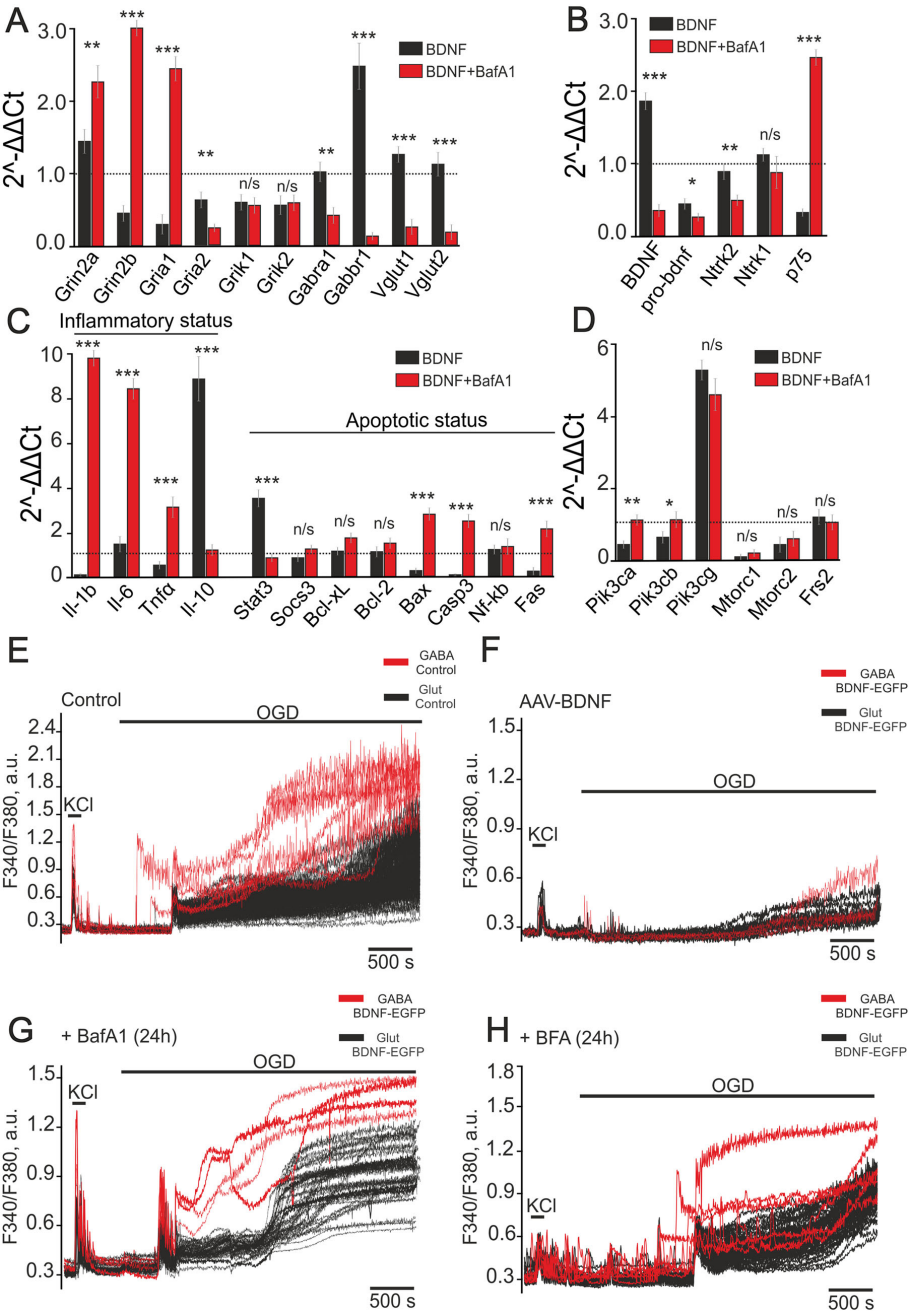
Effects of bafilomycin A1 on gene expression, changes of intracellular Ca^2+^ concentration under OGD, and OGD-induced cell death in (AAV)-Syn-BDNF-EGFP-transduced hippocampal cultures. **A**–**D** Effects of BafA1 on the expression of genes encoding glutamate transporters and subunits of the receptors (**A**); genes encoding BDNF and its precursor, pro-BDNF, and their receptors (**B**); genes regulating apoptosis and inflammation (**C**); genes encoding subunits of PI3K (Pik3ca, Pik3cb, and Pik3cg), mammalian target of rapamycin (Mtorc1 and Mtorc2) and fibroblast growth factor receptor substrate 2 (Frs2) (**D**) [expression of the selected genes in non-transduced cultures (control) set at 1 (dashed line); differences are relative to the BDNF group without BafA1 pre-incubation; *P*-values in text]. **E**–**H** OGD-induced Ca^2+^ responses of glutamatergic (black traces) and GABAergic (red traces) neurons from non-transduced cultures (Control, **E**), (AAV)-Syn-BDNF-EGFP-transduced hippocampal cultures (**F**, AAV-BDNF), (AAV)-Syn-BDNF-EGFP-transduced hippocampal cultures after 24-h pre-incubation with 1 µmol/L bafilomycin A1 (+BafA1) (**G**), and with the vesicular trafficking inhibitor, 50 µmol/L brefeldin A (**H**, +BFA).

We have previously shown that BDNF is released by hippocampal cells under OGD and glutamate toxicity [22], and others have reported the same results [32]. We showed that the release of BDNF is a Ca^2+^-dependent process that is suppressed by BafA1, a vacuolar ATPase inhibitor [22]. Incubation of (AAV)-Syn-BDNF-EGFP-transduced hippocampal cultures with BafA1 (1 µmol/L) for 24 h evoked a dramatic change in the basal expression of the genes encoding the vesicular glutamate transporters and subunits of GABA and glutamate receptors (Fig. 6A). Grin2a expression increased by 64% (*P *= 0.008), Grin2b by 731% (*P *≤ 0.001), and Gria1 by 737% (*P *≤ 0.001) compared to transduced cultures without BafA1 pre-incubation (Fig. 6A). It has been noted that the GluA2 subunit regulates the Ca^2+^ conductivity of AMPARs. Increased Gria1 expression was found along with suppressed Gria2 expression that probably contributes to the formation of Ca^2+^-permeable AMPARs. Gabra1 and Gabbr1 expression decreased by 60% (*P *= 0.003) and 94% (*P *≤ 0.001) after 24-h pre-incubation with BafA1. Interestingly, the levels of Grik1 and Grik2 expression were not affected by BafA1 (*P *= 0.1 and *P *= 0.11). Furthermore, Vglut1 and Vglut2 expression also decreased by 80% and 84% (*P *≤ 0.001 for both). However, the expression of the genes encoding kainate receptor subunits did not change in transduced cultures after pre-incubation with BafA1.

The expression of the genes encoding BDNF and pro-BDNF decreased by 81% (*P *≤ 0.001) and 47% (*P *= 0.017) after pre-incubation with BafA1, compared to the transduced cultures without BafA1 (Fig. 6B). The basal expression of Ntrk2 was significantly lower (44%, *P *= 0.005) while p75 expression was elevated by 668% (*P *≤ 0.001), which may stimulate the induction of apoptosis and necrosis.

Indeed, the expression of genes encoding pro-inflammatory cytokines was substantially higher [650% (*P *≤ 0.001) for IL-1β, 463% (*P *≤ 0.001) for IL-6, and 494% (*P *≤ 0.001) for TNFα] (Fig. 6C), while the expression of the IL-10 gene fell by 613% (*P *≤ 0.001). Basal expression of the anti-apoptotic gene Stat3 decreased by 317% (*P *≤ 0.001) after 24-h pre-incubation with BafA1, whereas expression of the pro-apoptotic genes Bax, Casp-3, and Fas increased by 190% (*P *≤ 0.001), 200% (*P *≤ 0.001), and 834% (*P *≤ 0.001), respectively. The elevated expression of pro-apoptotic genes points to the abolition of the anti-apoptotic effect of BDNF in the presence of BafA1. BafA1 did not significantly change the expression of the anti-apoptotic genes Socs3 (*P *= 0.47), Bcl-xL (*P *= 0.4), and Bcl-2 (*P *= 0.38).

Pik3ca and Pik3cb expression increased by 144% (*P *= 0.004) and 74% (*P *= 0.019) after 24-h pre-incubation with BafA1 (Fig. 6D). However, the expression levels of these genes in the transduced cultures were similar to those in the non-transduced cultures (Fig. 6D). BafA1 did not significantly change the expression of Pik3cg (*P *= 0.16), Mtorc1 (*P *= 0.21), Mtorc2 (*P *= 0.24), and Frs2 (*P *= 0.13).

**Fig. 7:**
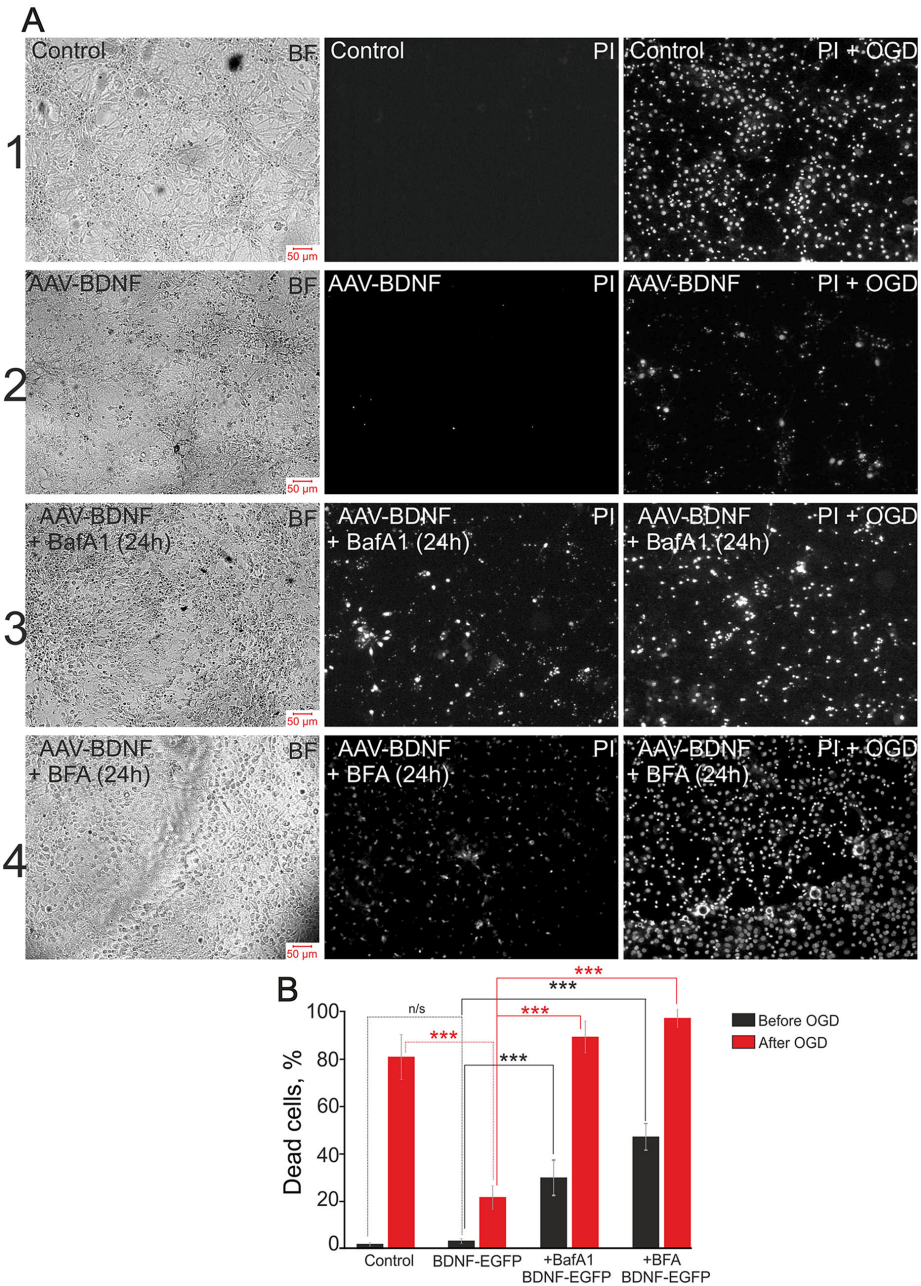
Viability of hippocampal cells after 40-min OGD in control and (AAV)-Syn-BDNF-EGFP-transduced cultures incubated with inhibitors of vesicular release. **A** Representative microphotographs of hippocampal cell cultures: bright-field images (left panels) and PI fluorescence (white dots) before (PI, central panels) and after OGD (PI + OGD, right panels). 1: Control, non-transduced cultures; 2: cultures transduced with (AAV)-Syn-BDNF-EGFP; 3: transduced cultures pre-incubated for 24 h with 1 µmol/L bafilomycin A1; 4: transduced cultures pre-incubated for 24 h with 50 µmol/L brefeldin A. **B** Average percentages of dead cells before (black) and after (red) 40-min OGD in control, (AAV)-Syn-BDNF-EGFP-transduced cultures, and (AAV)-Syn-BDNF-EGFP-transduced cultures with 24-h pre-incubation with 1 µmol/L bafilomycin A1 and 50 µmol/L brefeldin A (***P ≤ 0.001, n/s *P *= 0.28; vitality tests were made before and after OGD, see Fig. 6).

OGD evoked biphasic Ca^2+^ responses in glutamatergic and GABAergic neurons (Fig. 6E). The first phase occurred synchronously in glutamatergic and in most GABAergic neurons after a lag period, whose duration varied from culture to culture. It should be noted that some GABAergic neurons responded to OGD earlier than other GABAergic and glutamatergic neurons. The amplitudes of the [Ca^2+^]_i_ increase during the second phase of the OGD-induced Ca^2+^ response were higher for GABAergic than for glutamatergic neurons. Staining with PI showed 80% ± 16% dead cells after OGD, while PI fluorescence was detected only in individual cells before the experiments (Fig. 7A).

The first phase of the OGD-induced Ca^2+^ responses was significantly suppressed in glutamatergic and GABAergic neurons in the (AAV)-Syn-BDNF-EGFP-transduced cultures (Fig. 6F). The second phase (a global [Ca^2+^]_i_ increase) was also suppressed. The number of dead cells decreased dramatically due to this protective effect of BDNF overexpression in neurons. However, the number of dead cells after 40-min OGD in (AAV)-Syn-BDNF-EGFP-transduced cultures was 20% ± 6% (Fig. 7A, B). It should be noted that the protective effect of BDNF overexpression in neurons was abolished after 24-h pre-incubation of the transduced cells with BafA1. And a biphasic [Ca^2+^]_i_ elevation occurred in glutamatergic and GABAergic neurons (Fig. 6G). Furthermore, the proportion of dead cells after 40-min OGD rose to 91% ± 5% after pre-incubation with BafA1 (Fig. 7A, B). PI staining revealed that the percentage of dead cells in cultures with BDNF overexpression in neurons was 6% ± 4% before the experiments (Fig. 7A, B). Nevertheless, 33% ± 11% of dead cells were found before the experiments after 24-h pre-incubation with BafA1 (Fig. 7B).

Similar to BafA1, incubation of the (AAV)-Syn-BDNF-EGFP-transduced cultures for 24 h with BFA (50 µmol/L), an inhibitor of protein transport, abolished the protective effect of BDNF overexpression (Fig. 6H). Two phases of OGD-induced Ca^2+^ response were detected in glutamatergic and GABAergic neurons. The amplitude of the response during the first phase was lower compared to the incubation with BafA1. GABAergic neurons demonstrated increased OGD-induced Ca^2+^ activity (Fig. 6H) that can be a sign of hyperexcitation. BFA induced the death of 25% ± 12% of cells before the experiments, and this increased to 94% ± 6% (Fig. 7A, B) after 40-min OGD.

**Fig. 8:**
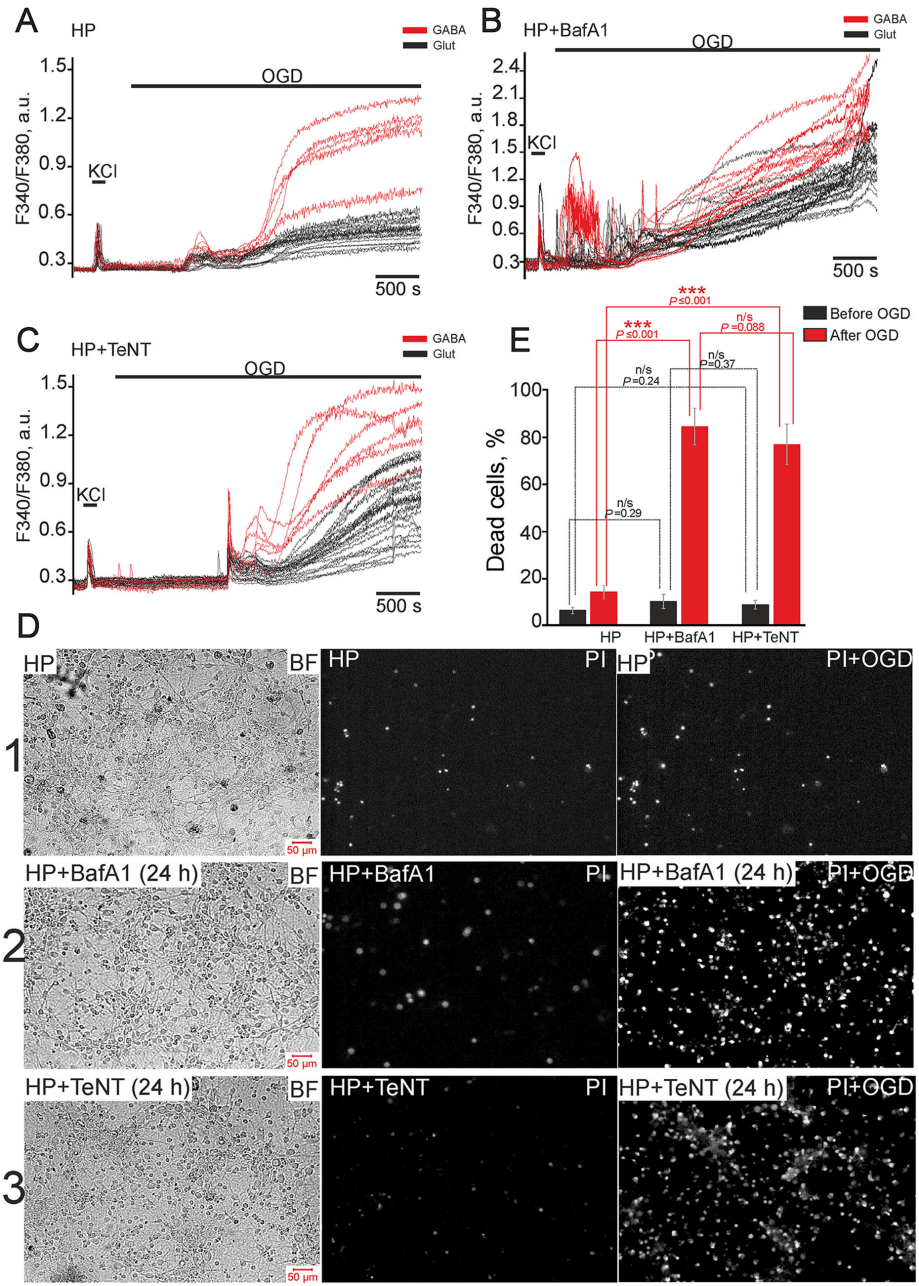
Suppression of vesicular release abolishes the protective effects of hypoxic preconditioning and promotes the death of hippocampal cells under OGD. **A**–**C** OGD-induced Ca^2+^ responses of glutamatergic (black traces) and GABAergic (red traces) neurons from cultures preconditioned with brief episodes of hypoxia/reoxygenation. (**A**) Controls (HP); cultures pre-incubated for 24 h with 1 µmol/L bafilomycin A1 (HP + BafA1) (**B**) and 50 ng/mL tetanus toxin (HP + TeNT) (**C**). **D** Representative microphotographs of hippocampal cultures: bright-field (left panels); PI fluorescence (white dots) before (central panels) and after OGD (right panels). 1: cultures preconditioned with HP; 2: preconditioned cultures pre-incubated for 24 h with 1 µmol/L BafA1; 3: preconditioned cultures pre-incubated for 24 h with 50 ng/mL TeNT. **E** Average percentage of dead cells before (black) and after (red) 40-min OGD in preconditioned cultures: control; pre-incubated for 24 h with 1 µmol/L BafA1 or 50 ng/mL TeNT. Vitality tests were performed before and after the OGD experiments in **A**, **B**, and **C**.

HP activated protective mechanisms predominantly in glutamatergic neurons (Fig. 3), thus promoting their survival under OGD. The experiments were performed using cell cultures preconditioned with repetitive hypoxia/reoxygenation episodes (Fig. 8). Cultures were divided into two groups after preconditioning. The first group was used as a control, while BafA1 (1 µmol/L) or TeNT (50 ng/mL) was added to the second group. The cultures were used in experiments 24 h after the preconditioning.

We found that the first and especially the second phase of OGD-induced Ca^2+^ responses were suppressed in glutamatergic neurons relative to control cultures (Fig. 8A) and the percentage of necrotic cells was reduced to 17% ± 6% (Fig. 8D, E). However, the amplitudes of the first phase of OGD-induced responses of GABAergic neurons (Fig. 8A) were higher than those in glutamatergic neurons and the responses of GABAergic neurons were characterized by high-amplitude irreversible elevation of [Ca^2+^]_i_ during the second phase.

In cultures pre-incubated with BafA1, high-amplitude Ca^2+^ responses in glutamatergic and GABAergic neurons appeared immediately after (without a lag-period) the application of OGD-medium (Fig. 8B), and high-amplitude Ca^2+^ oscillations occurred. The percentage of necrotic cells was 83% ± 8% (Fig. 8D, E). TeNT, an inhibitor of Ca^2+^-dependent vesicular fusion, also abolished the protective effects of HP. A high-amplitude, reversible [Ca^2+^]_i_ elevation appeared in glutamatergic neurons after a lag (Fig. 8C) and was followed by an irreversible global [Ca^2+^]_i_ increase 3–6 min later. Both phases of OGD-induced Ca^2+^ responses were also detected in GABAergic neurons after pre-incubation with TeNT (Fig. 8C). However, the amplitudes of signals during the second phase were higher in GABAergic than in glutamatergic neurons, and the second phase occurred earlier. The percentage of dead cells in the HP + TeNT group after OGD was 79% ± 7% (Fig. 8D, E), not significantly different from the HP + BafA1 group. It should be noted that the differences between the percentages of dead cells before OGD in the HP (8% ± 5%), HP + BafA1 (12% ± 6%), and HP + TeNT (10% ± 5%) groups were not significant (Fig. 8D, E).

**Fig. 9:**
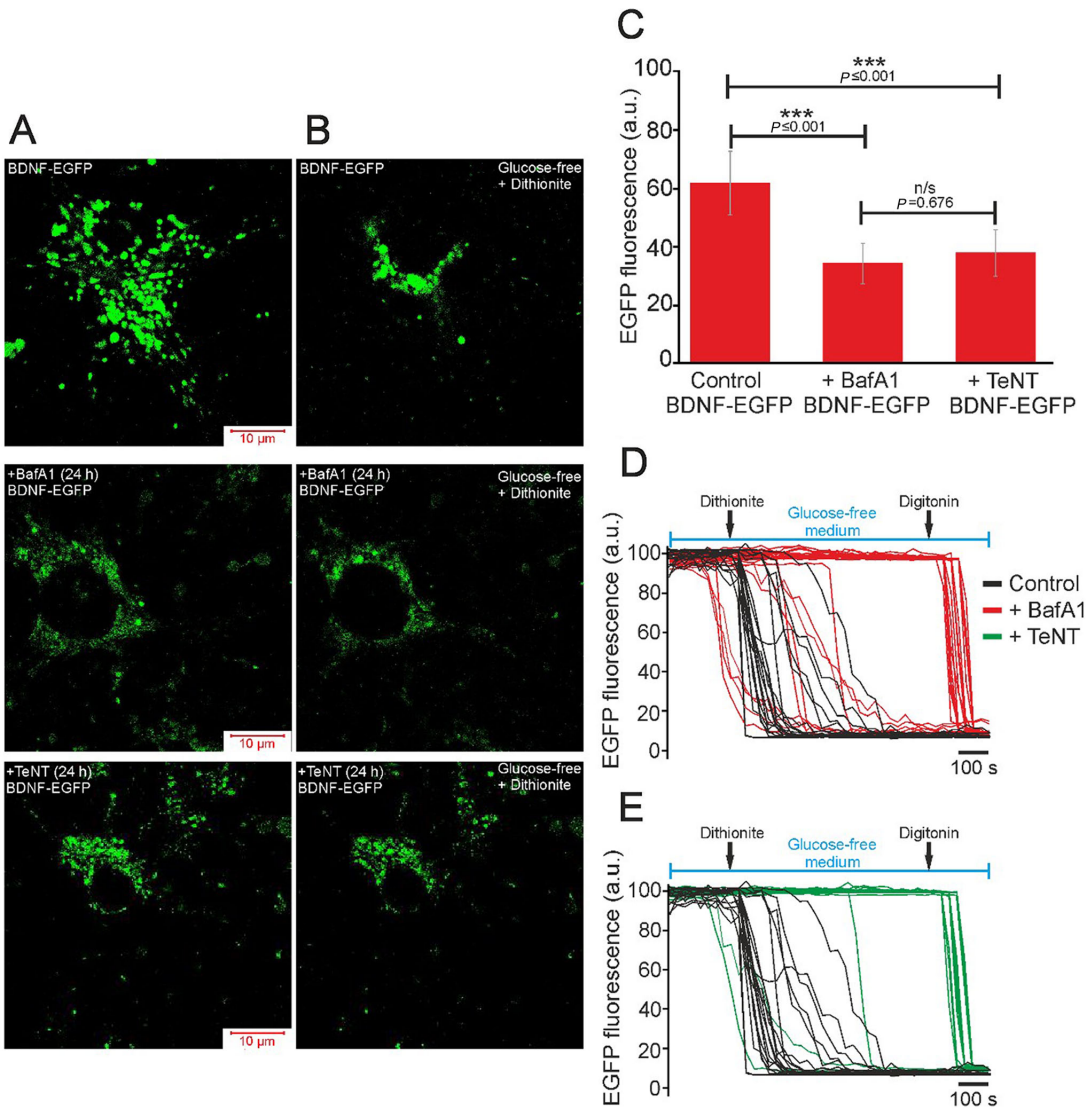
Effects of bafilomycin A1 (BafA1) and tetanus toxin (TeNT) on vesicular BDNF release under chemical OGD. **A**, **B** Images of individual representative neurons from a control cell culture transduced with the (AAV)-Syn-BDNF-EGFP construct (BDNF-EGFP) and a transduced cell culture pre-incubated for 24 h with BafA1 (1 µmol/L) or TeNT (50 ng/mL). The images were captured before (**A**) and after (**B**) 10-min chemical OGD (glucose-free medium + dithionite). All images were captured using the same settings of gain, laser power, and pinhole. **C** Average intensity of EGFP fluorescence in controls and 24 h after incubation with BafA1 or TeNT. **D**, **E** Plots showing a dithionite-induced decrease of BDNF–EGFP fluorescence intensity recorded by time-lapse confocal microscopy in individual neurons in control (black traces), and after 24-h pre-incubation with BafA1 (**D**, red traces) or TeNT (**E**, green traces). Decreased EGFP fluorescence was only found in cultures pre-incubated with BafA1 and TeNT after application of 3 µmol/L digitonin.

Using confocal microscopy, we established that the overexpressed BDNF was distributed in cells as individual vesicles demonstrating EGFP fluorescence (Fig. 9A). Addition of the O_2_ scavenger sodium dithionite to the glucose-free medium led to a rapid decrease in EGFP fluorescence intensity (Fig. 9B) and the disappearance of most BDNF-containing vesicles (Fig. 9D), indicating BDNF release under OGD. Incubation of (AAV)-Syn-BDNF-EGFP-transduced cultures for 24 h with 1 µmol/L BafA1 and 50 ng/mL TeNT (Fig. 9A) changed the number and size of vesicles. Basal EGFP fluorescence was significantly lower in these experimental groups than in controls (Fig. 9C). This finding indirectly indicated a decrease of the BDNF level in cells after pre-incubation with BafA1 and TeNT. The addition of dithionite to neurons pre-incubated with BafA1 induced the release of individual BDNF-containing vesicles (Fig. 9B, D), while the intensity of EGFP fluorescence decreased dramatically after application of the detergent digitonin. More pronounced suppression of dithionite-induced BDNF release was found in neurons from (AAV)-Syn-BDNF-EGFP-transduced cultures incubated with TeNT, an inhibitor of Ca^2+^-dependent vesicular fusion (Fig. 9B), and BDNF release was almost completely suppressed (Fig. 9E).

Thus, BafA1 and TeNT inhibited BDNF release from neurons transduced with (AAV)-Syn-BDNF-EGFP and affected the number and size of BDNF-containing vesicles. Suppression of vesicular secretion of BDNF abolished its protective action, and this was realized *via* the regulation of gene expression. The basal expression of genes encoding subunits of glutamate receptors increased substantially against the backdrop of reduced expression of the vesicular glutamate transporters and subunits of the GABA receptors. Furthermore, the expression of pro-inflammatory and pro-apoptotic genes also increased, indicating the induction of cell death by BafA1. The results of vitality tests, which were performed before the experiments, confirmed this conclusion (Fig. 7). These changes of gene expression may promote dysregulation of Ca^2+^ homeostasis in GABAergic neurons, leading to an irreversible global increase in [Ca^2+^]_i_ followed by death under OGD.

Thus, suppression of vesicular BDNF release abolished the protective effects of BDNF on hippocampal glutamatergic and GABAergic neurons under OGD, while the profile of gene expression turned towards apoptosis and excitotoxicity. Preconditioning of non-transduced cultures (without BDNF overexpression) with hypoxia/reoxygenation episodes promoted the development of resistance to OGD-induced damage. This effect of HP was expressed as decreases in the amplitudes of OGD-induced Ca^2+^ responses. However, we did not find this effect in GABAergic neurons. Moreover, the inhibitors of vesicular release abolished the protective action of HP in glutamatergic neurons. This finding indicated a pivotal role of vesicular release in the development of the protective effects of HP and the survival of glutamatergic neurons even in cultures without BDNF overexpression.

## Discussion

We demonstrated in the present study the mechanisms underlying the neuroprotective action of vesicular BDNF release by neurons transduced with an adeno-associated virus construct. These mechanisms involved changes of expression of genes that promote the activation of HP in GABAergic neurons and enhancement of this phenomenon in glutamatergic neurons. We showed that BDNF overexpression in neurons affected the conductivity of ion channels of glutamate receptors, the intensity of inhibitory neurotransmission, and the expression of pro- and anti-apoptotic genes, probably promoting cell survival under further prolonged episodes of O_2_ or O_2_-glucose deprivation.

It has been reported that the effects of hypoxia are dual or even in opposition. It has been demonstrated that rats exposed to hypoxia while sleeping have memory impairments caused by damage to specific populations of neurons in the hippocampus and cortex [33] due to hypoxia-induced apoptosis, oxidative stress, and endoplasmic reticulum stress [34]. Some of the effects of hypoxia on the gene expression in our experiments can be considered negative. We demonstrated in previous work that episodes of hypoxia-reoxygenation promote apoptosis in GABAergic neurons, while on the contrary, such episodes activate protective mechanisms in glutamatergic neurons [11]. Nevertheless, these effects were virtually suppressed in transduced cultures with BDNF overexpression in neurons. So, these negative effects can be explained by the suppression of BDNF synthesis in control cultures after hypoxia. It has been shown that BDNF is necessary for the consolidation of long-term synaptic plasticity [35, 36]. The expression of BDNF during chronic intermittent hypoxia decreases along with the expression of plasmin, which transforms pro-BDNF to BDNF; that is, BDNF synthesis is also attenuated. The application of BDNF restores the amplitude of long-term potentiation (LTP) in mouse hippocampal slices under hypoxia. Moreover, microinjection of BDNF into mice prevents the impairment of LTP [34].

It should be noted that the RNA used for PCR assay was extracted from all cells, including neurons and astroglia. However, the results of imaging experiments and vitality tests demonstrated that the protective effects of BDNF and HP, which promote the survival of GABAergic neurons, may be caused by the demonstrated changes in expression of the genes. Abolition of the protective effects of BDNF occurred upon the suppression of vesicular release and was accompanied by feedback changes of gene expression. Clearly, neuronal and neuroglial interactions can contribute to these effects and deserve investigation in further work. It is generally accepted that the damage and death of the most vulnerable populations of neurons under ischemia leads to excessive glutamate release and the intensification of brain injury. Activation of the preconditioning mechanisms can eliminate this secondary effect, which is caused by the death of cells. Glutamate excitotoxicity is mediated by the activation of glutamate receptors, primarily NMDARs and AMPARs. The expression of genes encoding subunits of NMDARs decreased significantly after episodes of hypoxia, whereas basal Grin2a expression was substantially higher. It has been reported that BDNF potentiates excitatory synaptic transmission in postsynaptic terminals by regulating the expression and trafficking of GluA1 subunits [37] as well as *via* the enhancement of GluN1, GluN2a, and GluN2B expression [38]. It has been shown that BDNF downregulates NMDAR function and decreases the NMDAR-mediated increase in [Ca^2+^]_i_ [39]. These findings agree with our results demonstrating decreased amplitudes of NMDA-induced Ca^2+^ responses in cultures with BDNF overexpression. Reduction of the number of GluA2-containing AMPARs under ischemia increases the vulnerability of neurons to glutamate excitotoxicity and Ca^2+^ overload [40]. A shift in the GluA1/GluA2 ratio is caused by a decrease of GluA2 expression [41]. In our experiments, the expression of both subunits was decreased in cultures with BDNF overexpression in neurons, indicating a reduction of the number of AMPARs in general. Increased Gria2 expression after hypoxia can be considered to be a neuroprotective effect.

It is known that the incubation of cells with BDNF does not alter the level of GAD65/67, the molecular marker of GABAergic neurons [42]. However, exogenous BDNF promotes the formation of inhibitory synapses in *in vitro* and *in vivo* models, while BDNF scavenging decreases the number of inhibitory synapses [43, 44]. In our experiments, increased expression of genes encoding GABA_A_ and GABA_B_ receptor subunits indicated the strengthening of inhibition in networks with BDNF overexpression and after episodes of hypoxia.

Interestingly, the expression of genes encoding the vesicular glutamate transporters Vglut1 and Vglut2 was elevated in the transduced cultures. Vglut1 expression increased further after episodes of hypoxia. Then, the expression of both genes decreased 24 h after episodes of hypoxia in non-transduced cultures. Vesicular glutamate transporters play a pivotal role in neurotransmission. The intensity of their expression correlates with synaptic strength [45], synapse formation, and the recycling of synaptic vesicles [46]. VGLUT1-deficient mice show decreased spontaneous glutamate release and suppressed quantal synaptic transmission in the hippocampus [44]. VGLUT1 overexpression enhances AMPAR-induced EPSCs *via* increasing the amount of glutamate in vesicles [47]. Decreased VGLUT1 and VGLUT2 expression changes the shape of secretory vesicles and their quantity in a synapse [46]. A deficit in VLUT2 suppresses glutamate secretion and attenuates long-term depression in synapses of the CA3–CA1 fields of the hippocampus in postnatal mice [48]. Decreased VGLUT1 expression causes memory impairment and depressive behavior [49], while VGLUT2-deficient heterozygous mice are characterized by neuropathic pain and defense responses [50]. The effects of BDNF on vesicular transporters have also been reported. It has been shown that BDNF enhances the expression of VGLUT1 and VGLUT2 *via* activation of Ntrk2 and PLCγ during the development of the hippocampus [42]. BDNF overexpression prevents the decrease of VGLUT1 expression and loss of glutamatergic synapses in a mouse model of Huntington’s disease [51].

Brief episodes of hypoxia promoted the increase of expression of PI3K subunits in both control and (AAV)-Syn-BDNF-EGFP-transduced cultures, and the activation of PI3K mediates many neuroprotective mechanisms. The PI3K/Akt signaling pathway plays a pivotal role in cell survival [52], the trafficking of synaptic proteins, and the regulation of protein synthesis [53]. The mTOR complex is one of the key proteins that mediate these effects [54]. BDNF/mTOR is involved in the modulation of autophagy and the maintenance of synaptic plasticity. Inhibition of mTOR causes autophagy-mediated degradation of AMPARs in spines [55]. Interestingly, the effects of hypoxia on the translation of proteins are mediated by the inhibition of mTORC1 [56], which is considered to be the main regulator of cell growth, proliferation, and protein synthesis [57]. Hypoxia-induced disturbances of mTORC1 regulation are coupled with cancerous growth [58]. It has been demonstrated that mTOR activation is necessary for angiogenesis under hypoxia and the proliferation of cells forming blood vessels [59]. On the one hand, hypoxia activates mTOR and stimulates angiogenesis [60], as well as the proliferation of lung fibroblasts and cells of the aortic wall [59], and increases the activity and concentration of hypoxia-inducible factor 1α [61]. On the other hand, hypoxia inhibits mTOR activity in the fibroblasts of mouse embryos and suppresses protein synthesis [62]. It has been shown that both mTORC1 and mTORC2 mediate the response of cells to hypoxia [63]. We showed that the expression of genes encoding mTOR and growth factor receptor substrate 2 (FRS-2) was suppressed after episodes of hypoxia. FRS-2 interacts with Trk proteins and activates the protective PI3K/Akt and MAPK cascades [64]. This finding can explain the decreased expression of many mTOR-regulated genes in control cultures after hypoxia. On the other hand, mTOR and Frs-2 expression increased in (AAV)-Syn-BDNF-EGFP-transduced cultures after hypoxia, thus promoting the development of HP and cell survival. It is known that the pro-inflammatory cytokine IL-1β suppresses the neuroprotective effects of BDNF and inhibits Akt [65]. Therefore, IL-1β can act as an endogenous inhibitor of the signaling pathway involving mTOR. In our experiments, Il-1β expression increased in control cultures after hypoxia, suggesting inhibition of mTOR by IL-1β. It has been demonstrated that the simultaneous application of IL-1β and BDNF or rapamycin (an inhibitor of mTOR) and BDNF causes cell death in a caspase-3-independent manner [66]. Therefore, mTOR activation is necessary for the realization of the neuroprotective effects of BDNF.

It is well-known that BDNF stimulates the expression of a range of anti-apoptotic genes and suppresses the expression of some pro-apoptotic genes. Overexpression of BDNF in hippocampal neurons treated with β-amyloid suppresses apoptosis *via* the activation of Bcl-2 expression and the prevention of cytosolic Ca^2+^ overload [67]. Activation of PI3K after preconditioning prevents cell death under ischemia *via* the suppression of Bax expression and the enhancement of Bcl-2 expression [68]. We demonstrated that the expression of anti-apoptotic genes such as Stat3, Socs3, and Bcl-xL increased in (AAV)-Syn-BDNF-EGFP-transduced cultures after hypoxia. In addition, the expression of genes encoding the anti-inflammatory cytokines IL-6 and IL-10 also increased in the transduced cultures 24 h after hypoxia. The same effect on the IL-10 level has been demonstrated after intranasal BDNF administration after ischemia [69].

Transcription of the BDNF gene is regulated by NF-κB and Ca^2+^ ions entering through ion channels and glutamate receptors [70–72]. Decreased levels of NF-κB and CREB have been demonstrated in models of post-traumatic stress disorder and “learned helplessness” [23]. We found decreased NF-κB expression in control cultures 24 h after hypoxia, while the expression of this gene was increased in transduced cultures with BDNF overexpression in neurons. Therefore, decreased NF-kB expression in controls can decrease the BDNF level.

Uncleaved pro-BDNF activates the high-affinity p75 receptor, which in turn induces pro-apoptotic signaling pathways [73]. Expression of the gene encoding pro-BDNF decreased in non-transduced cultures after hypoxia. Expression of Ntrk1 and Ntrk2 also decreased against the backdrop of elevated p75 expression. Mature BDNF activates tropomyosin-related kinase receptor type B (TrkB) [74] and the low-affinity neurotrophin receptor p75. The balance between the activity of these receptors determines the activation of signaling pathways promoting cell survival or cell death. Morphological changes and caspase-3 activation have been demonstrated in neurons with p75 overexpression three days after ischemia in the penumbra [75]. BDNF overexpression in neurons increased the Ntrk2 expression in our experiments. At the same time, p75 expression decreased substantially in the transduced cultures before as well as 24 h after hypoxia, thus preventing activation of the pro-apoptotic signaling cascades. Receptors for BDNF are expressed in glutamatergic and GABAergic neurons. BDNF is mainly localized in the presynaptic terminals of glutamatergic neurons during *in vitro* neurogenesis and maturation, while TrkB receptors are localized on both GABAergic and glutamatergic terminals. Interestingly, TrkB receptors are extrasynaptic and often co-localize with NMDARs and GABA_A_ receptors [76]. Selective knockout of TrkB receptors in corticolimbic GABAergic interneurons promotes the development of social dominance in male mice, while optogenetic inhibition of the excitatory activity of neurons normalizes social behavior [77]. TrkA and TrkB receptors are often localized in GABAergic and glutamatergic neurons [78], and their activation by nerve growth factor or BDNF has anti-apoptotic effects [79, 80]. It is known that the expression of BDNF and TrkA receptors in hippocampal neurons decreases after cerebral ischemia [81, 82], but the expression of TrkB receptors increases [82]. The expression of BDNF and its TrkB receptor recovers better in preconditioned hippocampal neurons [83]. The expression of genes encoding TrkA and TrkB receptors decreased after episodes of hypoxia-reoxygenation in our experiments. This decrease coincided with the decrease of BDNF expression that can be considered a negative effect. On the contrary, the expression of BDNF and its receptors increased in cultures with BDNF overexpression, thus promoting the activation of HP in GABAergic neurons and enhancing this phenomenon in glutamatergic neurons. Taking into account that BDNF modulates both excitatory and inhibitory neurotransmission, it can be concluded that the changes of TrkA and TrkB expression affect glutamatergic and GABAergic neurons.

The effects of BDNF are realized *via* vesicular secretion and further binding with its receptors [84]. All the protective effects of BDNF overexpression in our experiments were abolished after inhibition of vesicular transmission with BafA1 and BFA. Clearly, the increased expression of some genes after incubation of cultures with BafA1 cannot be explained only by suppressed vesicular BDNF release. It is evident that BafA1 affects other aspects of cell function. The best known and well-described effects of BafA1 are the induction of apoptosis in a caspase-independent manner and downregulation of Bcl-2 and Bcl-xL [85]. Nevertheless, we found that caspase-3, Bcl-2, and Bcl-xL expression was increased after 24-h pre-incubation with BafA1, indicating that the contribution of BafA1-induced apoptosis can be considered insignificant.

It is known that GABAergic neurons are sensitive to hypoxia. We showed in recent reports that elevation of [Ca^2+^]_i_ during brief episodes of hypoxia/reoxygenation occurs exclusively in GABAergic neurons. In addition, the percentage of necrotic cells 24 h after preconditioning is similar to that of GABAergic neurons in cell cultures [6, 11, 20]. It should be noted that mixed hippocampal cultures contain different cell types, including glutamatergic neurons, GABAergic neurons, and glial cells. In the present study, some conclusions were drawn based on data obtained as a result of non-selective analysis of large numbers of cells of the neuroglial complex (RT-PCR and vitality tests). The levels of expression of protective genes were higher in cultures with BDNF overexpression than in controls or preconditioned non-transduced cultures. In addition, OGD-induced necrosis and apoptosis were suppressed in transduced cultures, while pre-incubation of neuroglial cell cultures with inhibitors of vesicular release always enhanced the expression of pro-apoptotic and pro-inflammatory genes and increased the number of dead cells after OGD. Thus, most likely, BDNF overexpression also promotes the activation of neuroprotective signaling cascades in GABAergic neurons, because the percentage of living cells 24 h after 40-min OGD was ~ 100% (Fig. 4). All these data agree with the recordings of [Ca^2+^]_i_ dynamics in GABAergic neurons under OGD when BDNF overexpression promoted the development of the protective effects of HP and the suppression of global [Ca^2+^]_i_ elevation. Notably, preconditioning of non-transduced cultures with episodes of hypoxia induced the activation of protective signaling cascades and suppression of the OGD-induced Ca^2+^ responses only in glutamatergic neurons, thus indicating the protective effect of BDNF overexpression on GABAergic neurons.

Thus, we have demonstrated the critical role of BDNF in the development of cell tolerance to hypoxic conditions. Understanding the mechanisms of HP will help to determine pharmacological targets whose activation with endogenous or exogenous agonists may arrest the negative effects of global hypoxia or ischemia.

## Electronic supplementary material

Below is the link to the electronic supplementary material.

Supplementary material 1 (PDF 2449 kb)
