# Supplementary material for: BDNF Overexpression Enhances the Preconditioning Effect of Brief Episodes of Hypoxia, Promoting Survival of GABAergic Neurons
Source: Neurosci Bull. 2020 Mar 27;36(7):733–60. doi: 10.1007/s12264-020-00480-z (PMC7340710; doi:10.1007/s12264-020-00480-z)
Supplement: Supplementary file 1 — Supplementary material 1 (PDF 2449 kb) [file 12264_2020_480_MOESM1_ESM.pdf]

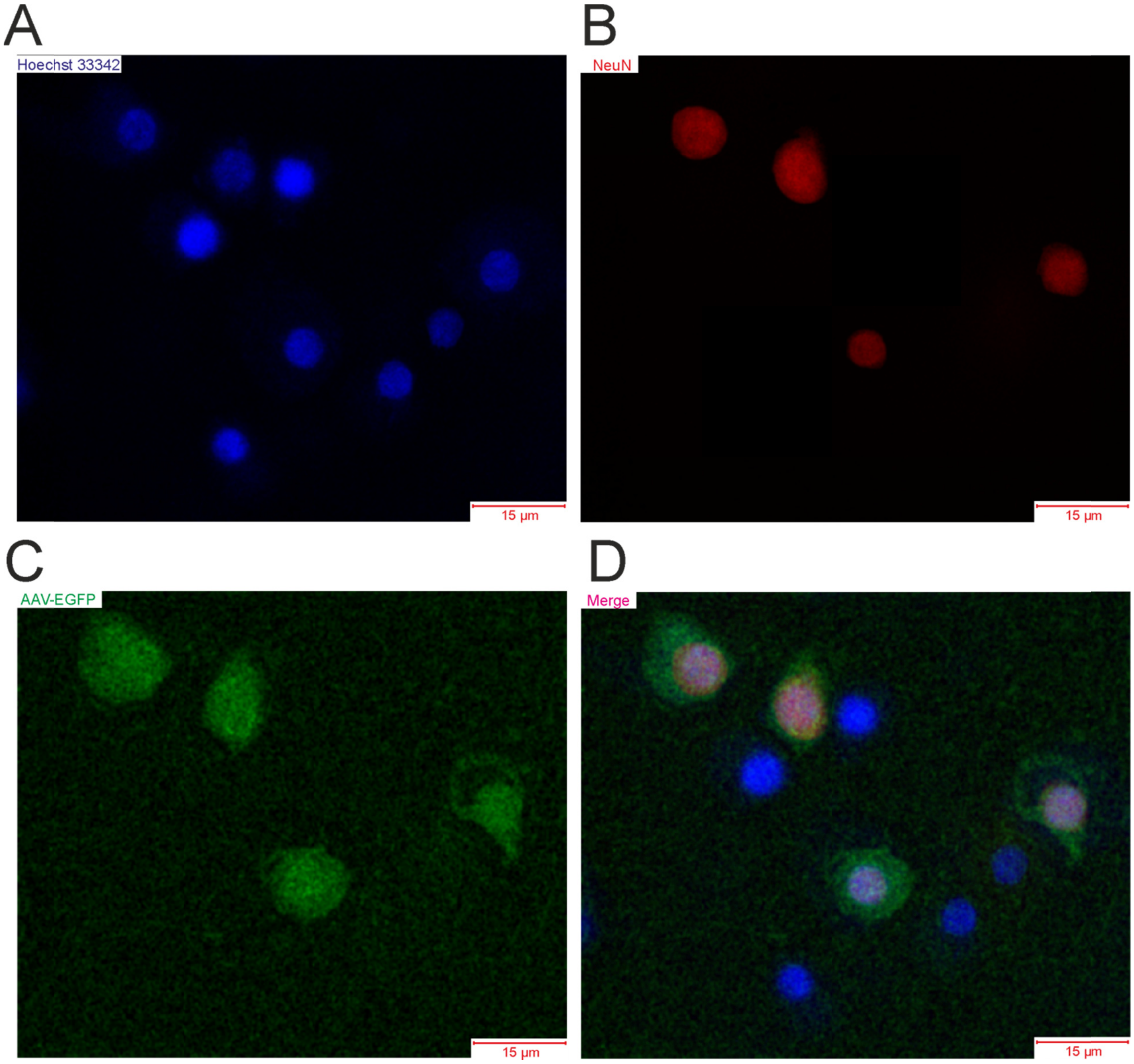

Fig. S1. Immunostaining of hippocampal cell culture transduced with (AAV)-Syn-BDNF-EGFP-construct. (A) Nuclei stained with Hoechst33342. (B) Fluorescence of antibodies against neuronal marker NeuN. (C) EGFP fluorescence in cells with BDNF overexpression. (D) Merge of Hoechst33342, NeuN and AAV-EGFP (BDNF) staining. From 9 cells in the view field, 4 cells are neurons. EGFP fluorescence was detected in all neurons.

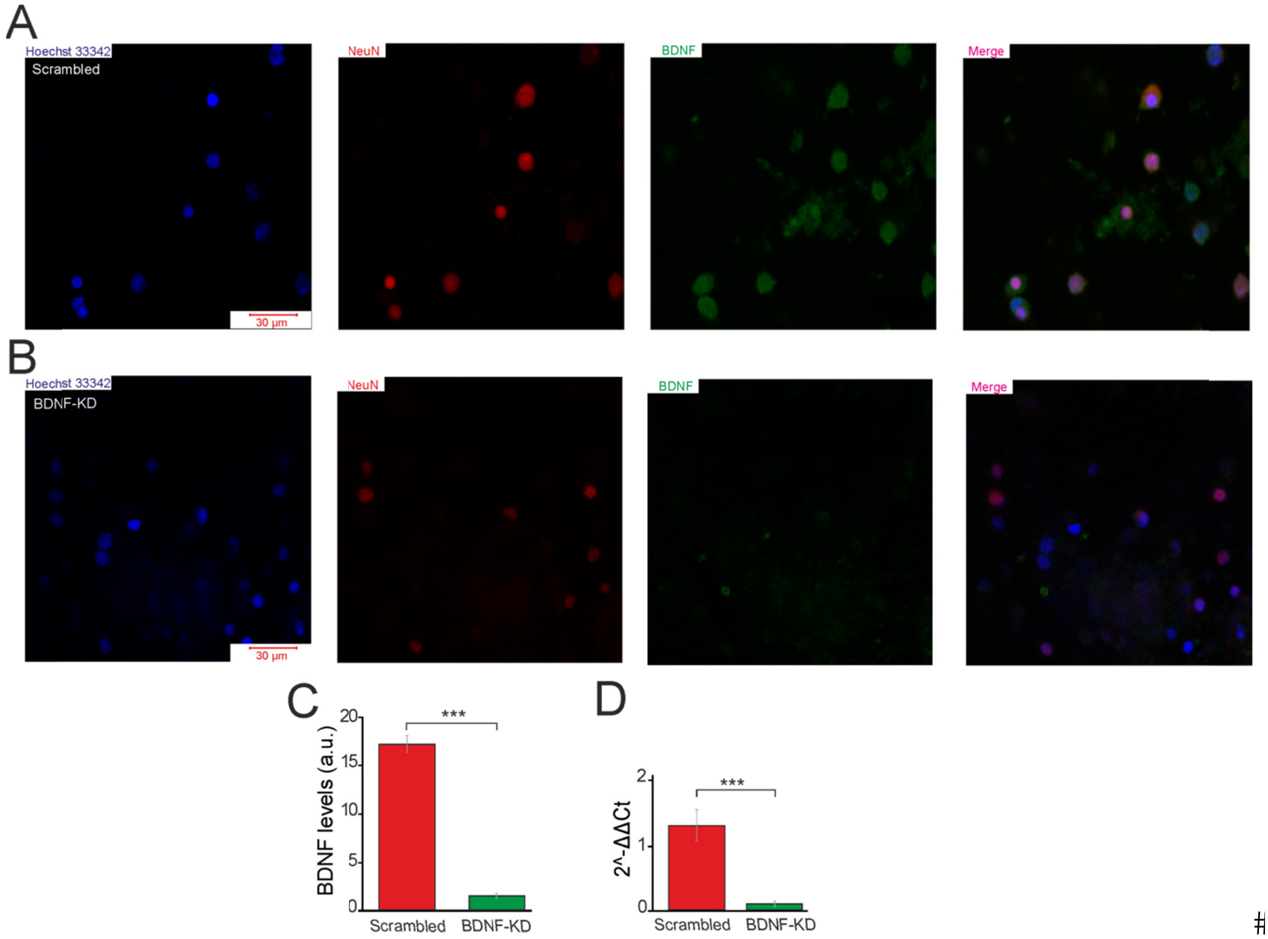

#

Fig. S2. The efficiency of BDNF knockdown. (A, B) Immunostaining of Scrambled cultures (A) and BDNF-knockdown cultures (BDNF-KD) (B) with antibodies against NeuN (red color) and BDNF (green color). Nuclei were stained with Hoechst 33342 (HO343) (blue color). (C, D) Effect of BDNF knockdown on the protein level (C) and mRNA expression of BDNF (D). The level of intracellular-expressed BDNF was determined by confocal imaging. We analyzed individual neurons with the fluorescence of Alexa Fluor 488 (BDNF, green color). The scrambled group (cell cultures transfected with shRNA) was used as control. The quantitative data reflecting the level of BDNF expression are presented as fluorescence intensity values in summary bar charts (mean ± SE). Statistical significance was assessed using paired *t*-test. We used the scans from three independent view fields for each experimental group. Values of each column were the average of 150 cells. The changes of expression were detected with RT-PCR assay. The expression in non-transfected cultures (control) was considered as 1. The cultures which were used in these experiments were not preconditioned with episodes of hypoxia. $^{***}P \leq 0.001$. #

#

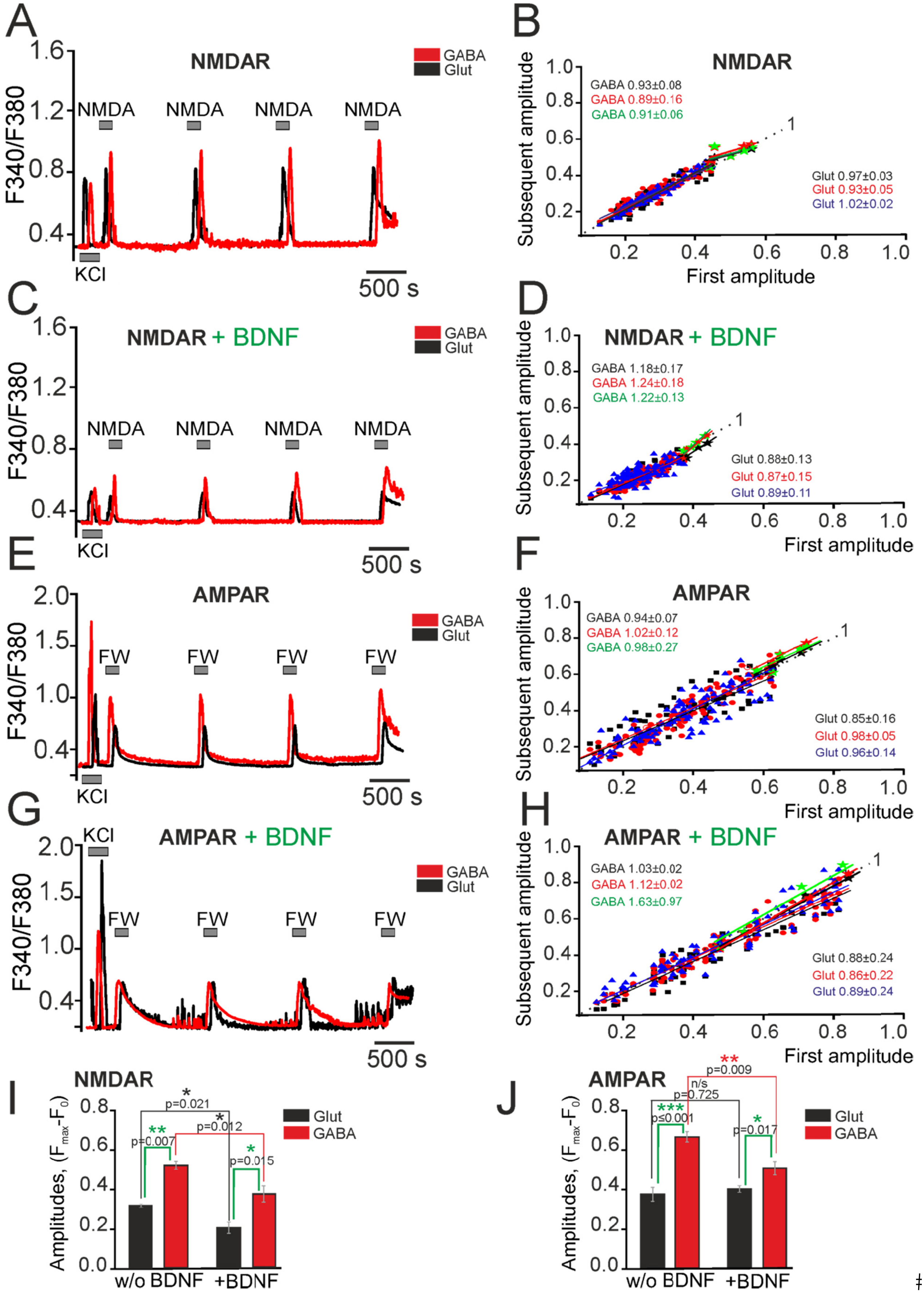

Fig. S3. Effects of BDNF overexpression on Ca^{2+} responses of glutamatergic (Glut, black curves) and GABAergic (GABA, red curves) neurons to repetitive applications of agonists

of AMPA and NMDA receptors in control cultures (without hypoxia). (A, C) Averaged Ca^{2+} responses of GABAergic (red curve) and glutamatergic (black curve) neurons non-transduced (A) and transduced with (AAV)-Syn-BDNF-EGFP construct (C), to repeated NMDA applications (10 µmol/L in Mg^{2+}-free medium). (B, D) The ratio of the amplitudes of Ca^{2+} responses of neurons to the second, third, and fourth NMDA application to the amplitudes of the response to the first NMDA application. X-axis, amplitudes of the responses to the first NMDA application; Y-axis, the amplitudes to the further NMDA applications. (E, G) Averaged Ca^{2+} responses of GABAergic (red curve) and glutamatergic (black curve) neurons non-transduced (E) and transduced with (AAV)-Syn-BDNF-EGFP construct (G), to repeated FW applications (0.3 µmol/L). (F, H) The ratio of the amplitudes of Ca^{2+} responses of neurons to the second, third, and fourth FW application to the amplitudes of the response to the first FW application. X-axis, amplitudes of the responses to the first FW application; Y-axis, the amplitudes to the further FW applications. (I, J) Averaged amplitudes of Ca^{2+} responses of glutamatergic (black columns) and GABAergic (red columns) neurons to NMDA (10 µmol/L in Mg^{2+}-free medium) and FW application in non-transduced (w/o BDNF) and transduced (+BDNF) cell cultures. In B, D, F and H, the dashed black line (1) demonstrates linear approximation whose slope value is 1. Black squares, red circles and blue triangles correspond to amplitudes of the Ca^{2+} responses of individual glutamatergic neurons to the second, third and fourth applications of NMDA or FW. Amplitudes of GABAergic neurons are marked with black, red and green star-shaped markers for the second, third and fourth applications of the agonists respectively. The colored values in panels are the slopes of linear regressions approximating Ca^{2+} responses of GABAergic (GABA) and glutamatergic neurons (Glut) to the applications of NMDA or FW. The color of values corresponds to the color of approximating lines.#

Table S1. Primer sequences for real-time polymerase chain reaction (RT-PCR).

**Name**	**Sequences**
Grin2a	Forward 5'-gctgacaaggatccgacatccacg-3'<br>Reverse 5'-gcccacaaagctgttgtccactgt-3'
Grin2b	Forward 5'-ggtgaggtggtcatgaagagggc-3'<br>Reverse 5'-gggttctgcacaggtacggagttg-3'
Gria1	Forward 5'-tgtctacatttatgatgctgaccggggc-3'<br>Reverse 5'-cgaggatgtagtggtacccgatgc-3'

Gria2	Forward 5'-gagggctactgtgttgacttagctgc-3'<br>Reverse 5'-5'-cctggtttggacttctgaggcttcttg-3'
Grik1	Forward 5'-ggaggatgaggcggggacc<br>Reverse 5'-gcatgctcttcgggaggcttcaaaac
Grik2	Forward 5'-ggatgggaaatatggagcccaggatgat<br>Reverse 5'-tcaggggagagaggattcaggaaggag
Gabra1	Forward 5'-tatctttgggcctggaccctcattctg-3'<br>Reverse 5'-ccataaggttgtttagccggagcactg-3'
Gabbr1	Forward 5'-tcctgtggaagaagaacagggggag-3'<br>Reverse 5'-gagtcaagccacggtacctgatgc-3'
Vglut1	Forward 5'-ggggaggctgcaccggttac<br>Reverse 5'-ggagccatgtatgaggccgacagt
Vglut2	Forward 5'-ccgagagaccatcgagctgacagag<br>Reverse 45'-gcagccagtcgcgatgcgatgtat
Pik3ca	Forward 5'-ctgagatgggagctgggactgc-3'<br>Reverse 5'-tagtttagtgctatcaaaccctgcttgcgtg-3'
Pik3cb	Forward 5'-gaggttatgagtgtgcttccgccctat-3'<br>Reverse 5'-agtcttcgtgtttcgtcttccagttcctc-3'
Pik3cg	Forward 5'-gctgcggagttctaccaccgattg-3'<br>Reverse 5'-tcagggaggtgagctgcttctgg-3'
Mtorc1	Forward 5'-gcaggtggatgccacagtgttca<br>Reverse 5'-cgagagttcgaagggcaagagtgatgc
Mtorc2	Forward 5'-gatgatggggaggtggacacggat<br>Reverse 5'-gcagaactcccaggcattctggct
Frs2	Forward 5'-gtgggaagcccgcaagctca<br>Reverse 5'-gccccccttccaagtccacctg
pro-Bdnf	Forward 5'-tcgaagagctgctggatgaggacc<br>Reverse 5'-gacccactcgctaatactgtcacacacg
Ntrk1	Forward 5'-ttcacccctcggctcagtca<br>Reverse 5'-agtcctgtagggagaggccc
Ntrk2	Forward 5'-gcactgtcctgctaccgcagttg<br>Reverse 5'-cagtcagtcagagtgcgtgtccctac
p75	Forward 5'-gcggagagtgctgcaaagcct<br>Reverse 5'-tcgtctgagtatgtgccctctggg

Il1b	Forward 5'-gaagcagctatggcaactgtccctga-3'<br>Reverse 5'-caggtcgtcatcatcccacgagtcac-3'
Tnfa	Forward 5'-ccgagatgtggaactggcagaggag-3'<br>Reverse 5'-ttggccaggagggcgttgg-3'
Il10	Forward 5'-tgggactgatgttgttgacagccact-3'<br>Reverse 5'-ccaggtagaaacggaactccagaagac-3'
Stat3	Forward 5'-cagctggacacgcgctacctg-3'<br>Reverse 5'-ctgcttctccgtcactacggcag-3'
Socs3	Forward 5'-cgtgcgccatggtcaccca-3'<br>Reverse 5'-gctgcccccctcgcact-3'
Bcl-2	Forward 5'-tggagatgaagactccgcgcccctga-3'<br>Reverse 5'-cgtggcaaagcgtcccctcgcggt-3'
Bcl-xl	Forward 5'-gagcagacccagtgagtgagcaggt-3'<br>Reverse 5'-ggggctatccgccaggtgc-3'
Casp3	Forward 5'-tcagaggcgactactgccggag-3'<br>Reverse 5'-cgtgagcatggacacaatacacgggt-3'
Il6	Forward 5'-tgggactgatgttgttgacagccactg-3'<br>Reverse 5'-ccaggtagaaacggaactccagaagacc-3'
Fas	Forward 5'-gtttggagttgaagaggagcgttcgt-3'<br>Reverse 5'-cattggcacactttcaggacttggg-3'

Note: Grin2a, glutamate ionotropic receptor NMDA type subunit 2A; Grin2b, glutamate ionotropic receptor NMDA type subunit 2B; Gria1, glutamate ionotropic receptor AMPA type subunit 1; Gria2, glutamate ionotropic receptor AMPA type subunit 2; Grik1, glutamate ionotropic receptor kainate type subunit 1; Grik2, glutamate ionotropic receptor kainate type subunit 2; Gabra1, gamma-aminobutyric acid type A receptor alpha1 subuni; Gabbr1, gamma-aminobutyric acid (GABA) B receptor, 1; Vglut1, vesicular glutamate transporter 1; Vglut2, vesicular glutamate transporter 2; Pik3ca, phosphatidylinositol-4,5-bisphosphate 3-kinase catalytic subunit alpha; Pik3cb, phosphatidylinositol-4,5-bisphosphate 3-kinase catalytic subunit beta; Pik3cg, phosphatidylinositol-4,5-bisphosphate 3-kinase catalytic subunit gamma; Mtorc1, mammalian target of rapamycin complex 1; Mtorc2, mammalian target of rapamycin complex 2; Frs2, fibroblast Growth Factor Receptor Substrate 2; pro-BDNF, precursor of Brain-derived neurotrophic factor; Ntrk1 (TrkA-receptor), tropomyosin receptor kinase A; Ntrk2 (TrkB-receptor), tropomyosin receptor kinase B; Il1b, interleukin 1 beta; Il10, interleukin 10; Tnfa, tumor necrosis factor a; Stat3, signal transducer and activator of transcription 3; Socs3,

suppressor of cytokine signaling; Bcl-2, b-cell lymphoma 2; Bcl-xl, b-cell lymphoma-extra large; Casp3, caspase-3; Il6, interleukin 6; Fas, tumor necrosis factor receptor superfamily, member 6.
